# Measuring audience experience across 20 showings of the same theatre performance using customer feedback kiosks and a questionnaire

**DOI:** 10.1371/journal.pone.0344225

**Published:** 2026-10-22

**Authors:** Mikko Sams, Einat Amir-Ahola, Enrico Glerean, Sirpa Kähkönen, Taru Mäkelä, Reetta Ristimäki, Antti Ruotoistenmäki, Veli-Matti Saarinen

**Affiliations:** 1 MAGICS–Aalto, Aalto University, Espoo, Finland; 2 Department of Neuroscience and Biomedical Engineering, Aalto University, Espoo, Finland; 3 The Faculty of Human and Social Sciences, Åbo Akademi University, Turku, Finland; 4 Aalto Scientific Computing, Aalto University, Espoo, Finland; 5 Greta Productions, Helsinki, Finland; 6 Aalto Studios, Aalto University, Espoo, Finland; 7 Aalto Behavioral Laboratory (ABL), Aalto Neuroimaging, Aalto University, Espoo, Finland; UKM: Universiti Kebangsaan Malaysia, MALAYSIA

## Abstract

Audience experiences can vary both between performances and among spectators, but little is known about **how different methods capture this variation across** repeated showings of the same production. We therefore examined audience experience across twenty live performances of the same music theatre production. We combined two complementary methods: immediate audience ratings collected through customer feedback kiosks and more detailed post-performance questionnaires. The kiosks recorded 1,887 responses, corresponding to 67% of total attendance, and 458 audience members (16%) completed questionnaires. Overall, the reception was positive, but kiosk ratings varied significantly across performances, indicating that audience-level enjoyment ratings differed from one showing to another. Kiosk and questionnaire responses to the same enjoyment question did not differ significantly at the aggregate level, suggesting that brief kiosk feedback may provide a practical, low-burden measure of audience-level enjoyment. Cluster analysis of questionnaire data identified three descriptive response profiles that differed primarily in overall engagement and ratings of specific artistic elements. These profiles illustrate heterogeneity within the audience of a single production. Qualitative responses from participants whose quantitative ratings were closest to each cluster centroid provided illustrative accounts of the experiences associated with these profiles. These narratives added context to the quantitative findings by showing differences in emotional tone, interpretive emphasis, and personal connection to the performance. Supplementary interviews with the director, artistic director, and an external playwright illustrated both interest in and reservations about incorporating audience data into creative work. Their comments emphasised the potential of empirical findings to support artistic reflection, while also underscoring the importance of how and when feedback is communicated within production teams.

## Introduction

Unlike recorded media, live theatre is a “people-oriented, intangible and perishable” [1] art form that takes place in the presence of an audience. Even when the same production is repeated, each performance is a distinct event: performers, audiences, and contextual conditions vary, and audience responses can therefore differ from one showing to another [2]. The audience is not merely a receiver. Bennett [3] argued that theatrical meaning is produced through the interaction between production and reception and is shaped by the social, spatial, and cultural setting. Audience responses are therefore part of the live event rather than only evaluations made after it.

The audience also contributes to the “co-creation of value” during a performance [4,5]. Spectators can influence one another through visible and audible reactions. Synchronised applause [6] and physiological entrainment among spectators [7], for example, show how an audience can temporarily respond as a collective. In this study, “collective audience experience” refers to the distribution and central tendency of individual audience responses within a given performance event. The term does not imply that all spectators shared the same subjective experience. Instead, it describes the audience-level response profile produced at each performance, which can be compared across repeated showings of the same production.

Audience experience includes emotional, social, intellectual, and artistic aspects. Audience studies have identified emotional stimulation as a common motivation for theatre attendance [8]. Emotional impact is a feature of memorable experiences [8], and empathy may contribute to transformative theatrical experiences [8]. Theatre attendance has also been described as a ritual that creates a shared setting and a sense of belonging [2]. These perspectives do not mean that spectators respond uniformly. Rather, they show why audience experience should be examined both at the level of individual spectators and at the level of the audience present at a particular performance.

Measuring this variation is difficult. Ticket sales, attendance figures, and critics’ reviews provide information about a production’s reach or reception but little direct evidence about spectators’ subjective experiences [9,10]. Post-performance interviews can allow spectators to explain their responses, whereas standardised questionnaires [11] can quantify dimensions such as engagement and emotional intensity [4]. Both require more effort from respondents and are difficult to collect at scale across many performances. Customer-feedback kiosks provide immediate, low-burden ratings [12], but usually address only a single overall judgement and do not explain why it was given. Repeated kiosk measurements can estimate variation between performances, whereas detailed questionnaires can reveal variation among spectators that an audience mean may conceal. No single method easily captures both levels.

Against this background, the present study had two linked aims. The scientific aim was to determine how much audience responses varied between 20 showings of the same music theatre production and among individual spectators, using immediate kiosk ratings and more detailed post-performance questionnaire data. We asked how these methods could be combined to characterise audience-level response patterns across repeated showings. Three principal analyses addressed this question. We examined whether kiosk enjoyment ratings differed across performances, whether kiosk and questionnaire responses to the same enjoyment question were comparable, and whether questionnaire responses revealed distinct experiential profiles among audience members. Associations with audience size and demographic factors were examined separately as exploratory analyses.

The practical aim was to examine how the resulting audience information might be interpreted and used in theatre work. The kiosk data served as an immediate measure of audience-level enjoyment, while the questionnaire provided more detailed individual ratings and open-ended responses. The open-ended responses were used to illustrate the experiential profiles identified in the questionnaire data. Interviews with theatre professionals were not treated as another measure of audience experience; they provided supplementary perspectives on the usefulness, limitations, and possible applications of audience data. This matters because audience measurement has limited practical value when its purpose, interpretation, and possible use are unclear to theatre professionals. The study was conducted collaboratively by academic researchers and theatre professionals so that the scientific questions and practical concerns of theatre work could be considered together.

## Methods

### The production

We measured audience experiences during 20 presentations of the music theatre performance “UKK”. The play is a carnivalesque story of Urho Kaleva Kekkonen (UKK), the president of Finland during 1956–1982. The plot draws on Finland’s cultural and political history, focusing on UKK’s role in managing Finland’s sensitive relationship with the Soviet Union and on his personality. He was a controversial figure, disliked by many but widely respected. The main events of the play occur in 1972–1973, when his presidency was extended without an election. The play was performed in Finnish; although language background was not assessed, most spectators were likely native Finnish speakers. The performance lasted 2 hours and 15 minutes, including an intermission between the two acts. The venue was a theatre “Kapsäkki” in Helsinki, Finland (https://kapsakki.fi/), which has a capacity of approximately 200 spectators. For further information about the production, please contact the corresponding author.

### Data collection

Immediately after each performance, audience members could voluntarily evaluate the performance using one of three customer-feedback kiosks. The kiosk screens displayed the question “How much did you enjoy the performance?” and six response buttons ranging from 1 (“not at all”) to 6 (“very much”). One kiosk was located beside the cloakroom, one on the route from the stalls, and one near the exit. At the end of every performance, the director, who also performed in the production, told the audience about the study and invited them to assist by rating the performance on the touchscreen kiosks and completing a short questionnaire accessed via QR code. Each button press was stored as an anonymous response. No personal or demographic information was collected through the kiosks, and kiosk responses could not be linked to questionnaire responses. The system did not restrict multiple button presses by the same person. Although repeated responses were considered unlikely in the busy exit setting, they cannot be ruled out. The kiosk hardware, data collection, and data storage were provided by PopUpBuddies (popupbuddies.fi).

Audience members were also invited to complete an online Finnish-language questionnaire administered using Webropol. No age restrictions or other eligibility criteria were applied; anyone who had seen the production could participate. Posters and leaflets containing a QR code were available in the foyer before the performance, during the intermission, and afterward. Respondents could access the questionnaire using their own mobile phone or computer, either at the theatre or later, and it remained open until the end of the production’s performance season. Respondents identified the performance they had attended, and this information was checked against the submission timestamp. The questionnaire contained 12 scaled items assessing different aspects of their experience of the performance. Each was rated on a six-point response scale with item-specific endpoint wording, with 1 corresponding to “not at all” and 6 to “completely.” These 12 items are shown in Fig 1 and formed the main questionnaire variables analysed in the present study. The academic researchers prepared an initial version of the questionnaire, which was then revised in consultation with the theatre professionals involved in the production.

**Fig 1:**
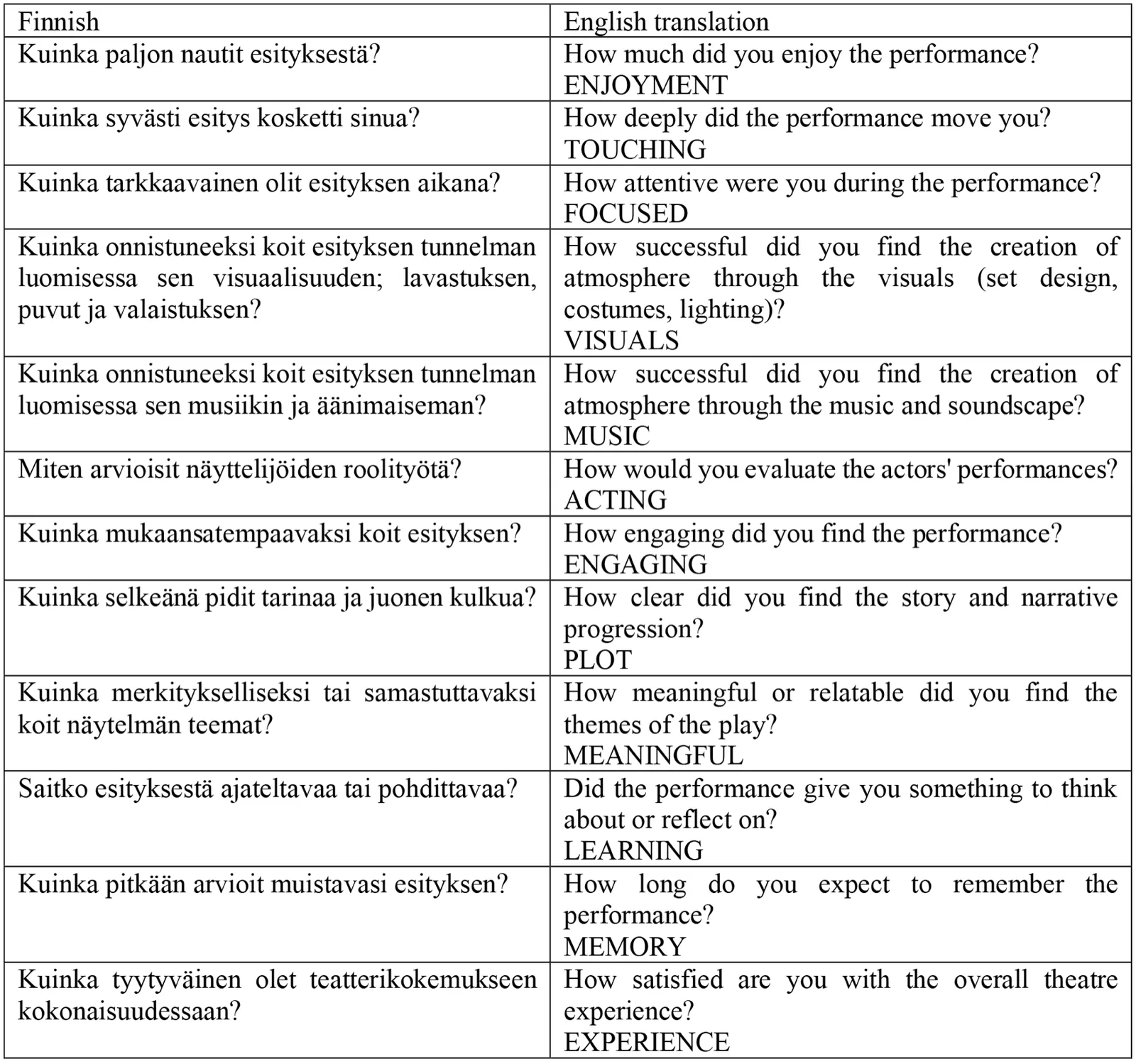
The 12 questionnaire items that formed the principal quantitative variables analysed in the present study. The original Finnish items, English translations, and abbreviated labels used in the analyses are shown. The complete questionnaire is provided in S1 Appendix (original Finnish version) and S2 Appendix (English translation).

Before beginning the questionnaire, respondents read an opening statement explaining the purpose of the survey, encouraging candid responses, and stating that questionnaire answers could not be connected to contact details supplied separately for the ticket draw. Proceeding to the questionnaire after reading this information indicated consent to participate. Questionnaire responses were anonymous. No direct compensation was provided, although respondents could enter a draw for free tickets by providing an email address on a separate form that could not be linked to their questionnaire responses.

The questionnaire also asked how likely audience members were to recommend the performance to others, using a 1–10 scale; this item was not analysed in the present study. In addition, it collected gender, age, theatre-going frequency, and an open-ended description of the respondent’s experience. Immediately after the scaled questions, optional question 14 allowed an unrestricted free-text response: “You may write, at whatever length you wish, about anything that remained in your mind from the performance. Was there anything in particular that stayed with you? Please use the following structure: 1) First half, 2) Second half.” Gender, age, and theatre-going frequency were used in the exploratory demographic analyses, and the open-ended responses were used for the illustrative cluster descriptions and sentiment analysis. The complete Finnish questionnaire is provided in S1 Appendix, and its English translation in S2 Appendix.

Questionnaire items were not compulsory, and partially completed questionnaires were retained. Analyses used available cases except where complete cases or imputation were specified in the data-analysis procedures below. The platform did not prevent repeat submissions. One incomplete record was removed after the respondent reported being unable to complete the questionnaire on the first attempt and then submitting another response. The same spectator could contribute both a kiosk rating and a questionnaire response, and audience members were encouraged to do so; however, responses from the two methods could not be linked.

Following the primary audience data collection, the second author (EAA), a researcher in interdisciplinary collaboration and artistic research, conducted individual semi-structured interviews with three theatre professionals who were also coauthors of the study: the director of UKK; the artistic director of the production, who was also a cast member; and a Finnish playwright who was not involved in the production. The interviews were conducted in English during September and October 2025. The director and artistic director were interviewed online via Zoom, and the playwright was interviewed by telephone. Each interview lasted approximately one hour. A common interview guide addressed the interviewees’ professional backgrounds, existing approaches to audience engagement and feedback, possible uses and risks of audience data, modification of productions during a performance run, audience–performer interaction, collaboration with researchers, and future applications of audience research in live theatre. All interviews were audio-recorded with written informed consent and transcribed verbatim. The transcripts were reviewed to identify statements directly relevant to the interpretation and practical application of audience data. Quotations were selected because they clearly illustrated recurring concerns or contrasting professional perspectives; no formal qualitative coding or thematic analysis was conducted. The interviews were used as supplementary professional perspectives rather than as an independent qualitative dataset. All interviewees approved the use of attributed quotations, reviewed the manuscript before submission, verified the accuracy of their quotations, and confirmed their consent for publication.

### Data analysis

For the kiosk data, we calculated the relative distribution of the six rating categories and the mean enjoyment rating for each of the 20 performances. Differences in rating distributions across performances were tested using a chi-square test of homogeneity, with Cramér’s V as the effect-size measure. The association between audience size and the mean kiosk enjoyment rating for each performance was examined using Pearson correlation and simple linear regression.

For each questionnaire item, we calculated the distribution of responses across respondents. To examine variation between performances, we first calculated the mean rating for each item separately for each performance and then calculated the mean, standard deviation (SD), and standard error of the mean (SEM) across the 20 performance-specific means. Differences in across-performance variance among the 12 questionnaire items were evaluated using both Levene’s test and the non-parametric Fligner–Killeen test. Kiosk and questionnaire responses to the shared enjoyment question were compared using a two-sided Mann–Whitney U test because the ratings were ordinal, the sample sizes differed, and individual kiosk and questionnaire responses could not be linked. Except for the cluster and demographic analyses, analyses used available cases; consequently, the number of observations varied slightly when an individual item response was missing. One questionnaire respondent did not provide an enjoyment rating, resulting in 457 questionnaire responses in the kiosk–questionnaire comparison.

Relationships among the 12 questionnaire items were examined using pairwise Pearson correlations. To characterise heterogeneity in questionnaire response profiles, the 12 item scores were standardised to zero mean and unit variance. Missing item values used in the cluster analysis were replaced with the mean of the corresponding item, allowing all 458 questionnaire respondents to be retained. We applied k-means clustering to the standardised 12-dimensional data and examined solutions containing one to ten clusters. The number of clusters was selected using the elbow method applied to the within-cluster sum of squared errors. The final three-cluster solution was calculated using 50 initialisations, a maximum of 1,000 iterations, and a fixed random seed of 42.

Principal component analysis (PCA) was performed on the same standardised questionnaire data. PCA was used to visualise and interpret the cluster solution and did not determine cluster membership. The clusters were numbered from Cluster 1 to Cluster 3 according to their mean score on the first principal component. To select illustrative open-ended responses, we calculated each respondent’s Euclidean distance from the centroid of their cluster in the standardised 12-dimensional questionnaire space. From each cluster, we selected the three respondents closest to the centroid who had also provided an open-ended response.

Associations between questionnaire ratings and demographic factors were examined in a complete-case sample of 437 respondents. Respondents were included if they had valid responses for gender, age, and theatre-going frequency and complete ratings for all 12 questionnaire items. Gender was analysed as a two-level categorical factor; respondents who selected “other” or did not report gender were therefore excluded. Because the youngest and oldest age categories contained few respondents, the < 20-year category was merged with the 21–30-year category, and the > 80-year category was merged with the 71–80-year category, resulting in six age groups. Theatre-going frequency was analysed as a six-level categorical factor. For each questionnaire item, gender, age group, and theatre-going frequency were examined separately using one-factor ordinary least-squares models followed by Type II analysis of variance. These analyses were exploratory. Within each demographic factor, the p values from the 12 questionnaire-item analyses were adjusted separately for multiple comparisons using the Benjamini–Hochberg false-discovery-rate procedure.

The open-ended responses were written in Finnish and automatically translated into English using a Python script. The translations were reviewed by Antti Ruotoistenmäki and Mikko Sams and corrected where necessary. We then quantified the emotional tone of the 268 checked English translations using the rule-based VADER (Valence Aware Dictionary and sEntiment Reasoner) algorithm implemented in NLTK version 3.9.1 [13]. VADER relies on a human-curated lexicon in which words, emoticons, and slang expressions have been assigned valence scores by trained raters. It also applies deterministic rules for negations, intensifiers, emoticons, and punctuation emphasis and produces a normalised compound polarity score ranging from −1 to +1 for each comment.

Although VADER enables rapid and reproducible sentiment measurement across a large set of comments, it cannot capture the full nuance of qualitative audience responses. Furthermore, VADER was developed for general-purpose English-language social-media text rather than theatre-specific research. Words carrying negative affect in everyday contexts, such as tragic or threatening, may refer to positively evaluated qualities in a theatre context, which limits the interpretation of the sentiment scores.

The distributions of VADER scores within the three clusters were examined using Shapiro–Wilk tests. Because the scores were non-normally distributed, differences among the clusters were tested using the Kruskal–Wallis test. A significant overall difference was followed by pairwise two-sided Mann–Whitney U tests with Bonferroni correction for the three cluster comparisons.

All statistical tests were two-sided, and the significance level was set at p < .05. Analyses were conducted in Python 3.11.13 using NumPy, pandas, SciPy, scikit-learn, statsmodels, and NLTK.

The audience data collection was conducted in accordance with the ethical principles of the Declaration of Helsinki and was approved by the Aalto University Research Ethics Committee (D/296/03.04/2022; updates in September 2023, October 2023, and August 2024). The later interviews with theatre professionals were not included in this ethics approval. All three interviewees provided written informed consent to participate and to be audio-recorded, approved the use of attributed quotations, reviewed the manuscript, verified the accuracy of their quotations, and confirmed their consent for publication. Separate ethical review was not required because the interviews concerned the participants’ professional views, involved no sensitive personal data, and the interviewees participated as identifiable professional collaborators and coauthors.

## Results

### Participants

A total of 2,812 people attended 20 performances of UKK between October 10 and December 14, 2024. Attendance varied considerably across performances (Fig 2). Demographic information was collected only from audience members who completed the post-performance questionnaire.

**Fig 2:**
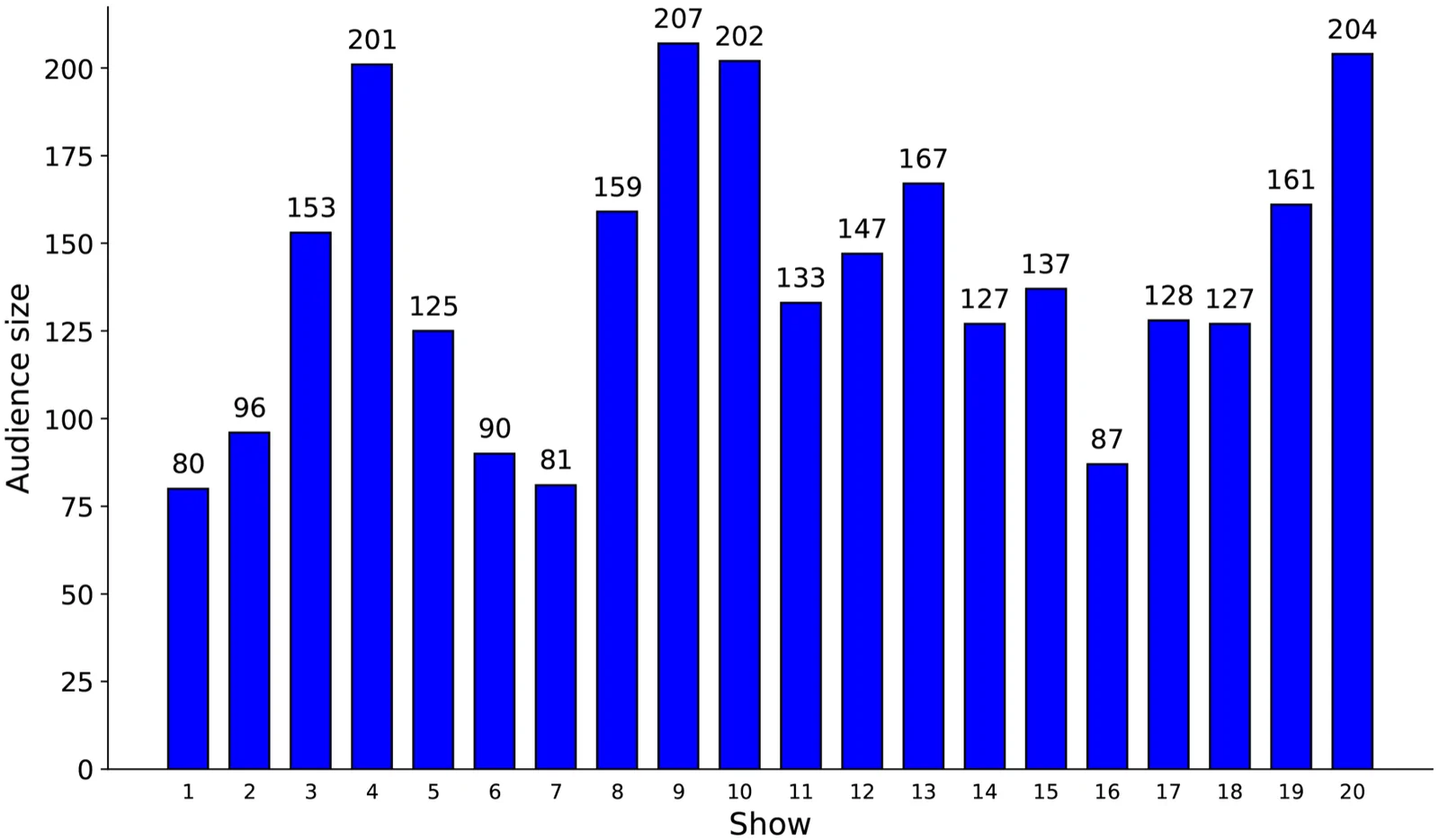
Audience sizes in all 20 performances, ordered according to the date and time of performance. Some of the performances were on the same day.

### Kiosk responses

The kiosks recorded 1,887 responses, corresponding to 67% of the total attendance. The distribution of the responses (mean across all kiosks) is depicted in Fig 3. The mean enjoyment rating was 4.50 (SD = 1.26, median = 5.00), indicating that the overall reception was strongly positive.

**Fig 3:**
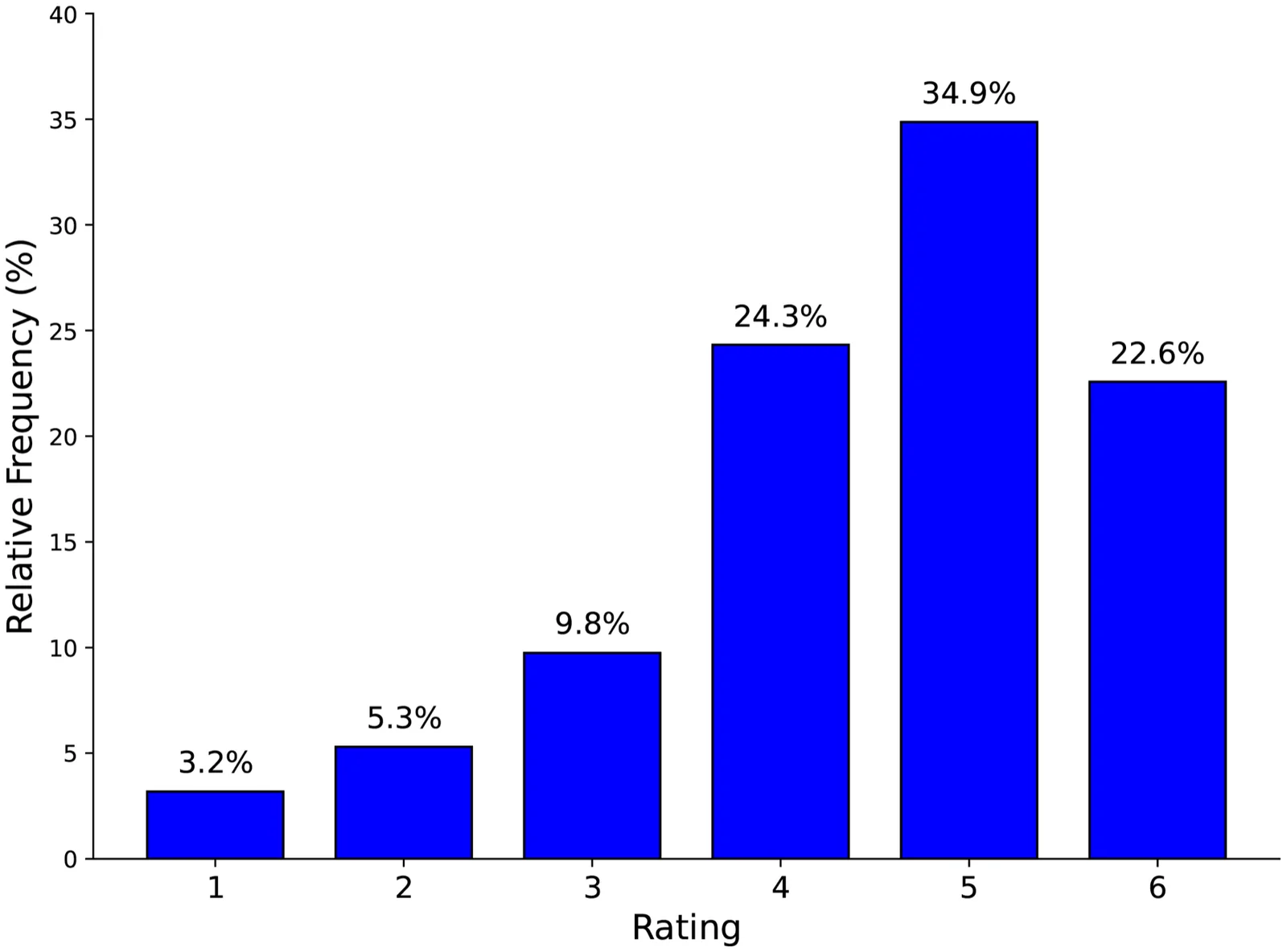
Relative frequencies of the kiosk responses. Respondents indicated their liking of the play on a scale 1–6 (not at all–very much).

The distributions of the kiosk responses varied considerably across the 20 performances (Fig 4). A chi-square test of homogeneity showed that the distribution of rating categories differed significantly across the performances (χ^2^(95, N = 1,887) = 249.32, p < .001, Cramér’s V = 0.21). We also examined whether the audience size was associated with the mean enjoyment ratings (Fig 5). This analysis showed that the audience size was negatively related to the mean kiosk enjoyment rating (Pearson r = −0.498, R^2^ = 0.248, t(18) = −2.436, p = .025). Although statistically significant, the effect was modest: the predicted mean enjoyment rating decreased from approximately 4.7 to 4.3 across the observed range of audience sizes.

**Fig 4:**
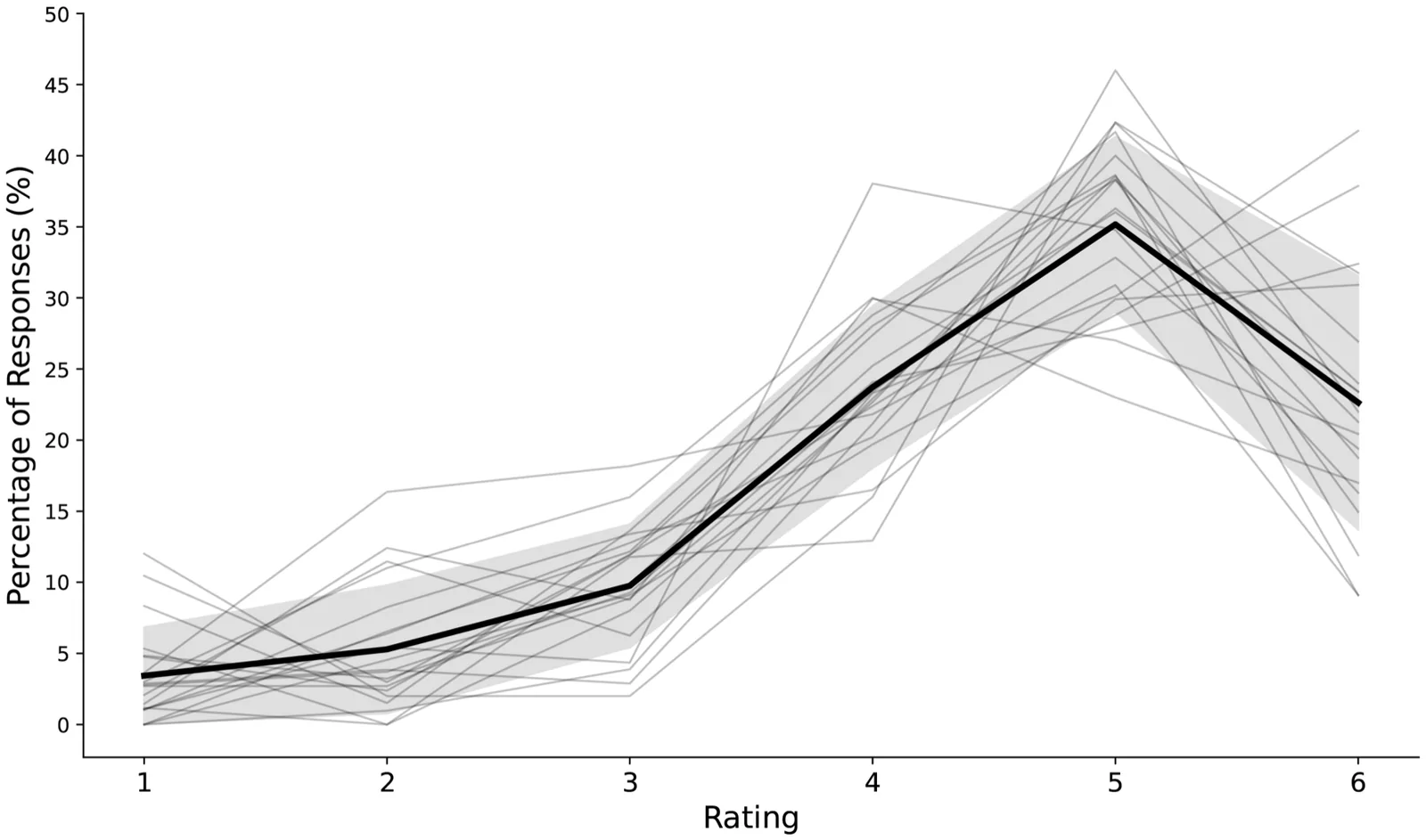
Relative frequencies of the six kiosk rating categories for each of the 20 performances. Thin lines show individual performances, the thick line shows the mean across performances, and the grey-shaded band indicates ±1 SD across performances for each rating category.

**Fig 5:**
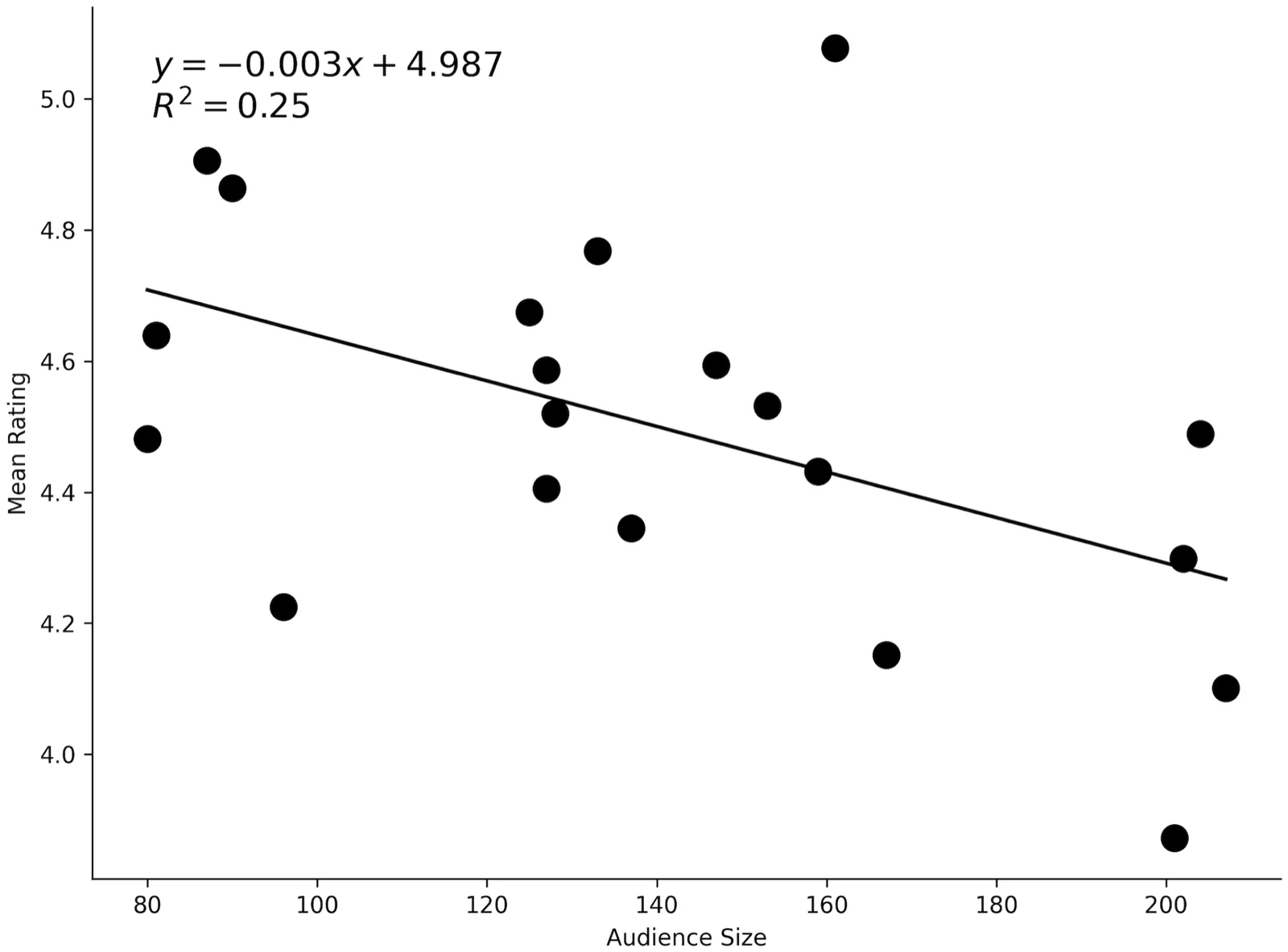
Relationship between audience size and mean kiosk enjoyment rating across the 20 performances. Larger audiences tended to give slightly lower mean ratings.

### Questionnaire responses

A total of 458 audience members completed the questionnaire, corresponding to 16% of total attendance (35% male, 64% female, three identified as other, and four did not report their gender). Fig 6 shows the age distribution of the questionnaire respondents. Among the questionnaire respondents, 78% were 51 years or older, and the largest age group was 61–70 years. This distribution differed substantially from that of the Helsinki region population, in which the corresponding proportions for the six middle age groups at the end of 2024 were 16%, 18%, 14%, 11%, 10%, and 8%, respectively. (https://stat.fi/tup/statfin/index_en.html).

**Fig 6:**
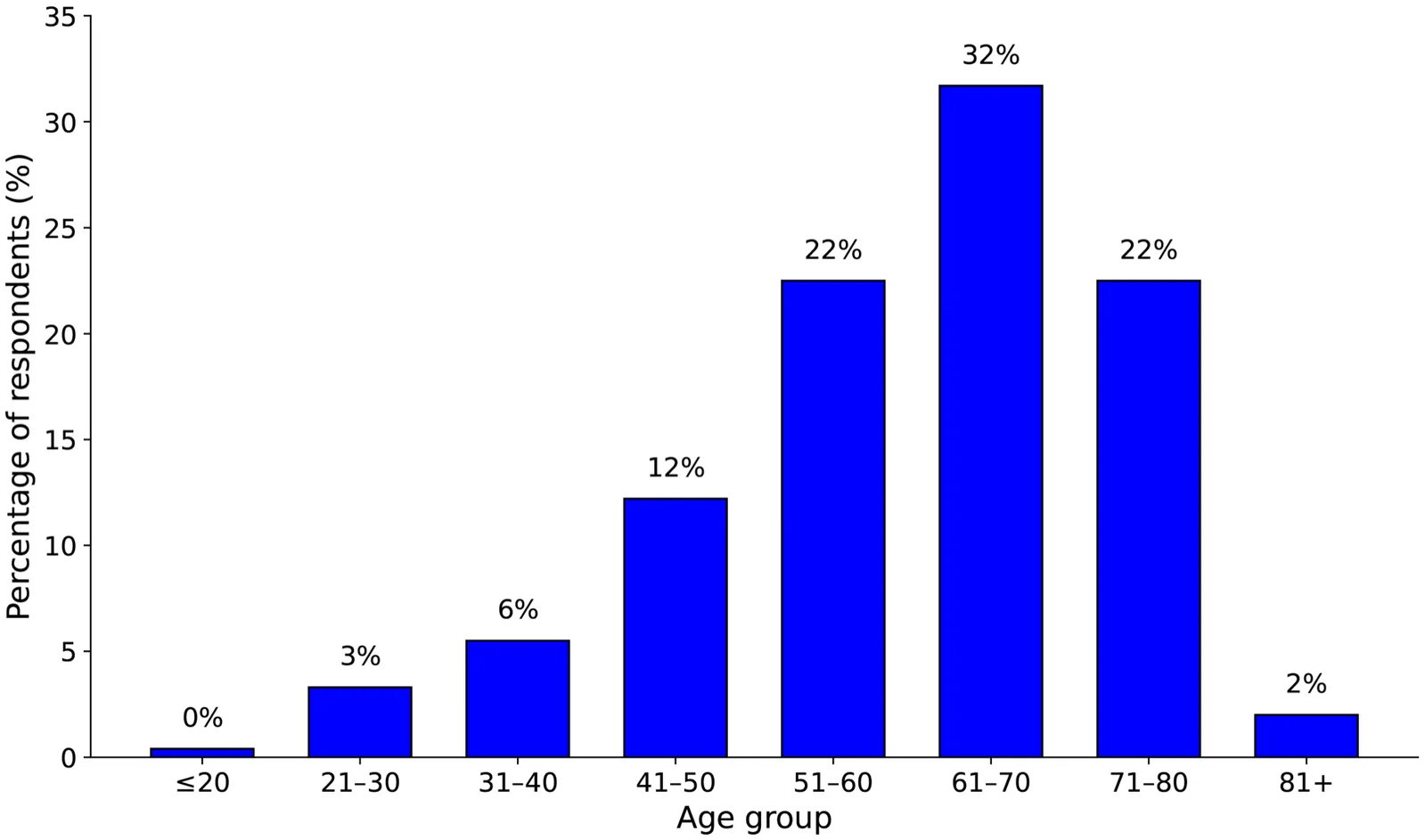
Relative age distribution of the questionnaire respondents. The x-axis shows the age group.

The relative distributions of how audience members answered the questionnaire are depicted in Fig 7. Responses to all questionnaire items were skewed toward the positive end of the scale. Mean scores for each questionnaire item are presented in Table 1.

**Fig 7:**
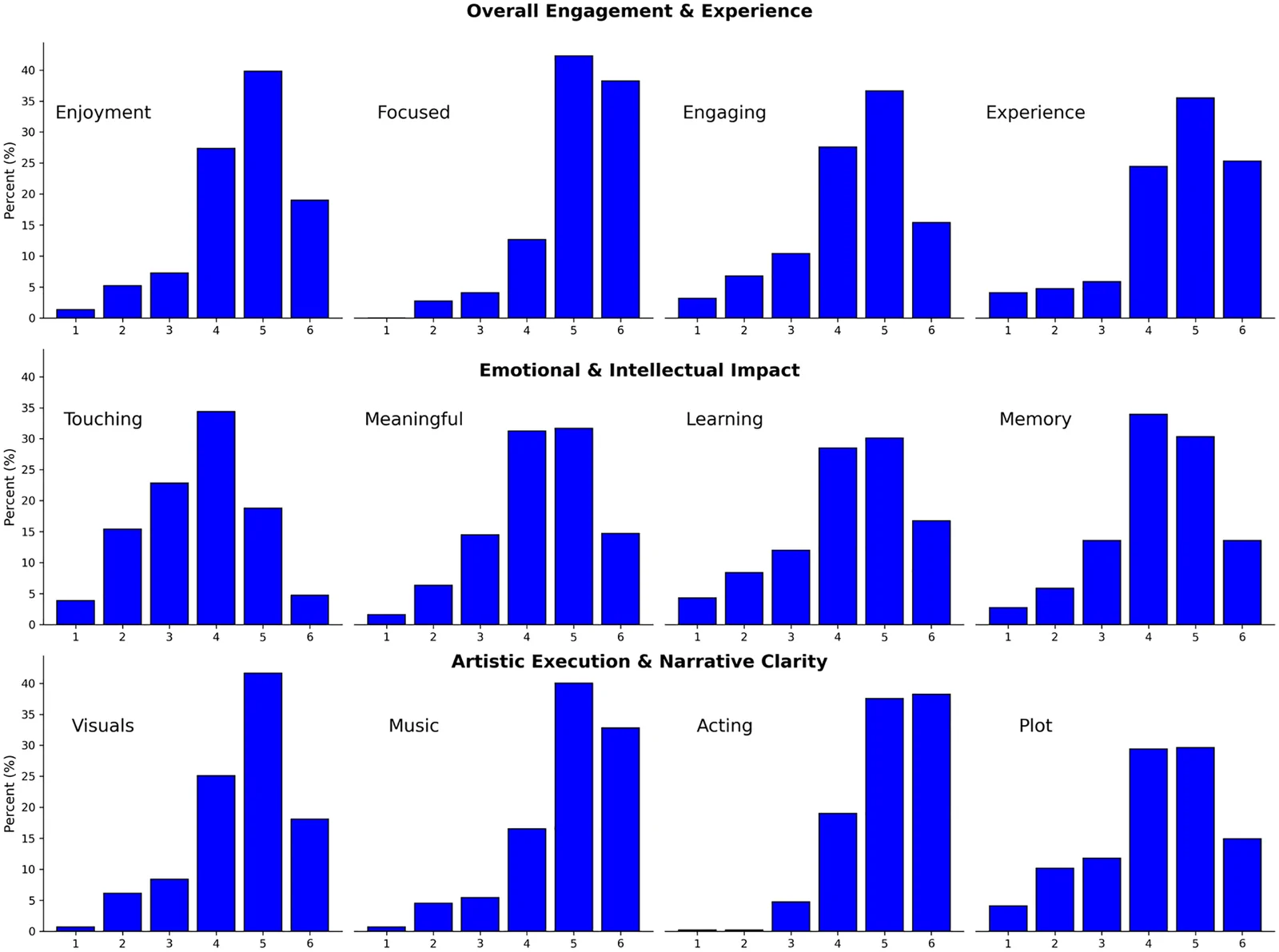
Relative distributions of questionnaire answers categorised into three groups. For questions, see Fig 1.

**Table 1:** Mean questionnaire ratings and variation across the 20 performances. Em dashes indicate that SD and SEM were not calculated for category means.

Questionnaire item	Mean	SD	SEM
**Overall engagement and experience**			
Enjoyment	4.58	0.28	0.06
Focused	5.10	0.28	0.06
Engaging	4.33	0.33	0.07
Experience	4.58	0.39	0.09
**Category mean**	**4.65**	**—**	**—**
**Emotional and intellectual impact**			
Touching	3.64	0.27	0.06
Meaningful	4.29	0.27	0.06
Learning	4.18	0.32	0.07
Memory	4.27	0.34	0.08
**Category mean**	**4.09**	**—**	**—**
**Artistic execution and narrative clarity**			
Visuals	4.56	0.31	0.07
Music	4.90	0.38	0.09
Acting	5.07	0.20	0.04
Plot	4.13	0.34	0.08
**Category mean**	**4.66**	**—**	**—**

For descriptive purposes, we grouped the questionnaire items into three conceptual categories. The first, “Overall Engagement and Experience,” comprised Enjoyment, Focused, Engaging, and Experience. Focused received the highest mean rating, while the other three items also received high ratings.

The second category, “Emotional and Intellectual Impact,” comprised Touching, Meaningful, Learning, and Memory. Touching received the lowest mean rating (3.64), while Meaningful, Learning, and Memory received mean ratings of 4.29, 4.18, and 4.27, respectively.

The third category, “Artistic Execution and Narrative Clarity,” comprised Visuals, Music, Acting, and Plot. Acting received the highest mean rating (5.07), followed by Music (4.90) and Visuals (4.56), whereas Plot received a lower mean rating (4.13).

Table 1 presents the across-performance mean, standard deviation (SD), and standard error of the mean (SEM) for each questionnaire item, together with the mean of the four items within each conceptual category. Average ratings ranged from 3.64 (Touching) to 5.10 (Focused), with most items receiving mean ratings between 4.1 and 5.1. Across-performance SDs ranged from 0.20 to 0.39, indicating modest variation in mean ratings between performances. Acting showed the smallest across-performance SD, whereas Experience showed the largest.

To test whether across-performance variance differed between questionnaire items, we used Levene’s test (W = 0.88, p = 0.563) and the non-parametric Fligner–Killeen test (χ² = 9.98, p = 0.532). Neither test was significant, providing no evidence that across-performance variance differed among the 12 questionnaire items. Although Experience showed numerically greater variability than some other items, these differences were compatible with sampling variation across the 20 performances.

To compare immediate kiosk ratings with post-performance questionnaire ratings, we examined responses to the same enjoyment question in both methods (Fig 8). A Mann–Whitney U test, appropriate for ordinal data and unequal group sizes, found no significant difference between kiosk and questionnaire ratings (U = 425,200, N(Kiosk) = 1,887, N(Questionnaire) = 457, p = 0.687).

**Fig 8:**
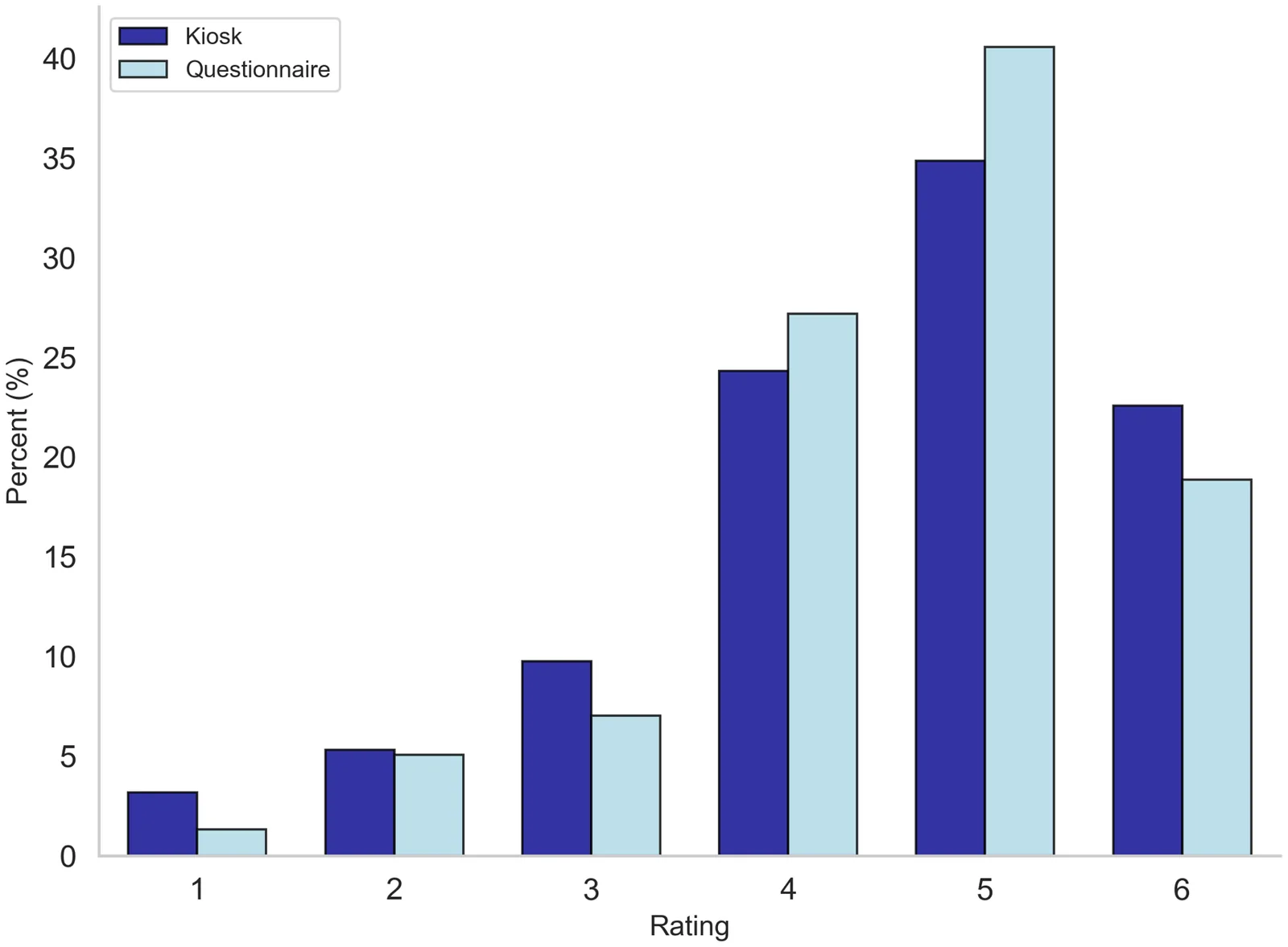
Relative distributions of the kiosk and questionnaire responses to the question “How much did you enjoy the performance?”.

To explore how different audience groups experienced the play, we conducted k-means cluster analysis on the standardised questionnaire responses. We then used principal component analysis (PCA) to visualise the resulting clusters in a two-dimensional space defined by the first two principal components, which together explained 66.3% of the variance (Fig 9). This provided an intuitive overview of how participants with similar rating profiles were distributed across the main dimensions of variation.

**Fig 9:**
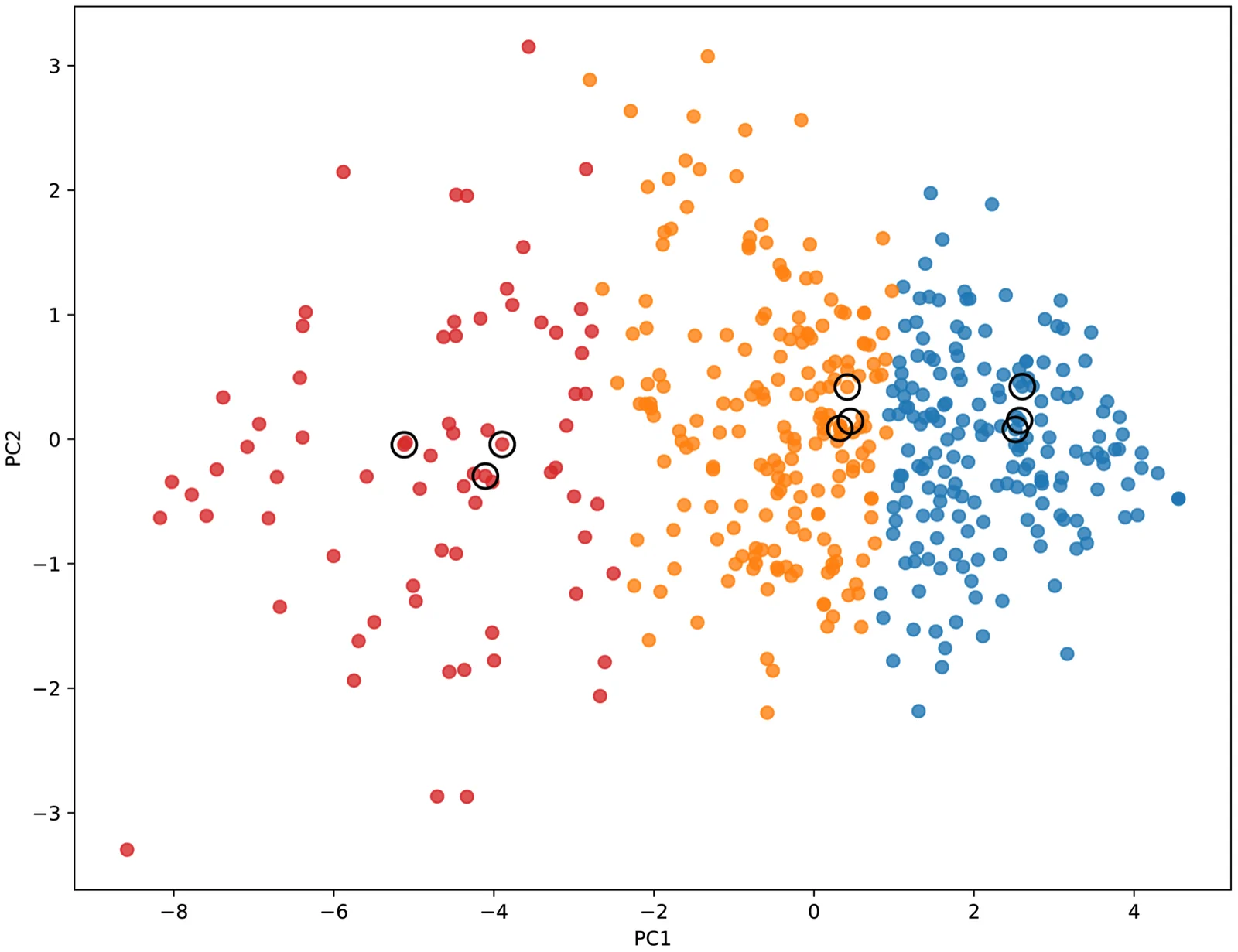
Visualisation of the k-means clusters in the space defined by the first two principal components. Each point represents one questionnaire respondent and is coloured according to cluster assignment: red indicates Cluster 1, orange Cluster 2, and blue Cluster 3. PC1 and PC2 together explained 66.3% of the total variance. Circled points indicate the three participants closest to each cluster centroid who also provided an open-ended response.

Each cluster comprised participants with similar response profiles across the 12 questionnaire items. The principal components were then used to interpret the main dimensions along which these profiles varied. PC1 explained 58.7% of the variance and was interpreted as an “Overall Engagement” dimension, with the highest loadings on Engaging (λ = 0.34), Experience (λ = 0.34), Enjoyment (λ = 0.33), and Touching (λ = 0.31). PC2 explained an additional 7.6% of the variance and showed its highest positive loadings on Music (λ = 0.50), Acting (λ = 0.38), and Visuals (λ = 0.37), while the loadings of the more emotionally oriented items were weaker or negative. We interpreted PC2 as a “Production and Artistic Quality” dimension (Table 2). Although it explained substantially less variance than PC1, it captured variation in evaluations of Music, Acting, and Visuals that was partly distinct from overall engagement.

**Table 2:** Loadings of the questionnaire items on the first two principal components and mean item ratings within the three participant clusters.

Questionnaire item	Principal component loadings		Mean rating by cluster		
	PC158.7%	PC27.6%	Cluster 1(n = 73)	Cluster 2(n = 197)	Cluster 3(n = 188)
Enjoyment	0.33	0.09	2.82	4.41	5.42
Touching	0.31	−0.24	2.08	3.36	4.53
Focused	0.21	−0.12	4.11	5.00	5.57
Visuals	0.28	0.37	3.05	4.48	5.23
Music	0.25	0.50	3.51	4.84	5.49
Acting	0.26	0.38	3.96	5.01	5.61
Engaging	0.34	0.09	2.30	4.21	5.30
Plot	0.27	−0.06	2.53	3.97	5.01
Meaningful	0.29	−0.37	2.86	4.03	5.13
Learning	0.28	−0.35	2.49	4.04	5.06
Memory	0.28	−0.32	2.71	4.02	5.08
Experience	0.34	0.08	2.41	4.50	5.54

The three rightmost columns of Table 2 show the mean rating for each questionnaire item within each cluster, together with the number of participants in each cluster. Cluster 1 showed the lowest mean ratings across most items, Cluster 2 showed intermediate ratings, and Cluster 3 showed the highest ratings. The clusters therefore primarily reflected graded differences in overall engagement and evaluation rather than three sharply distinct audience types.

We analysed gender (two levels), age (six ordinal groups; the two sparsely populated extreme categories were merged with the adjacent categories), and theatre-going frequency (six categories) as categorical factors in separate Type II ANOVAs for each of the 12 questionnaire items. No questionnaire item was significantly associated with gender or theatre-going frequency (all raw ps ≥ .05). After Benjamini–Hochberg correction across the 12 questionnaire items, associations with age group remained significant for Focused, Touching, Meaningful, Music, and Plot. The nominal association with Acting did not remain significant after correction (raw p = .045, FDR-adjusted p = .090; Table 3).

**Table 3:** Exploratory Type II ANOVA results for associations between age group and the 12 questionnaire items. The table shows the factor and error degrees of freedom, F statistic, raw p value, and Benjamini–Hochberg FDR-adjusted p value for each item. The two sparsely populated extreme age categories were merged with the adjacent categories.

Questionnaire item	Degrees of freedom		*F*	*p*	*p (FDR-adjusted)*
	Factor	Error			
**Overall engagement and experience**					
Enjoyment	5	431	1.059	.383	.417
Focused	5	431	4.047	.001	.005
Engaging	5	431	1.950	.085	.134
Experience	5	431	1.541	.176	.234
**Emotional and intellectual impact**					
Touching	5	431	4.885	<.001	.001
Meaningful	5	431	3.192	.008	.023
Learning	5	431	1.922	.090	.134
Memory	5	431	0.800	.550	.550
**Artistic execution and narrative clarity**					
Visuals	5	431	1.111	.354	.417
Music	5	431	2.729	.019	.046
Acting	5	431	2.288	.045	.090
Plot	5	431	7.132	<.001	<.001

Fig 10 shows mean ratings across age groups for all 12 questionnaire items. The clearest age-related trends were observed for Touching and Plot. In contrast, mean Music ratings decreased slightly with increasing age.

**Fig 10:**
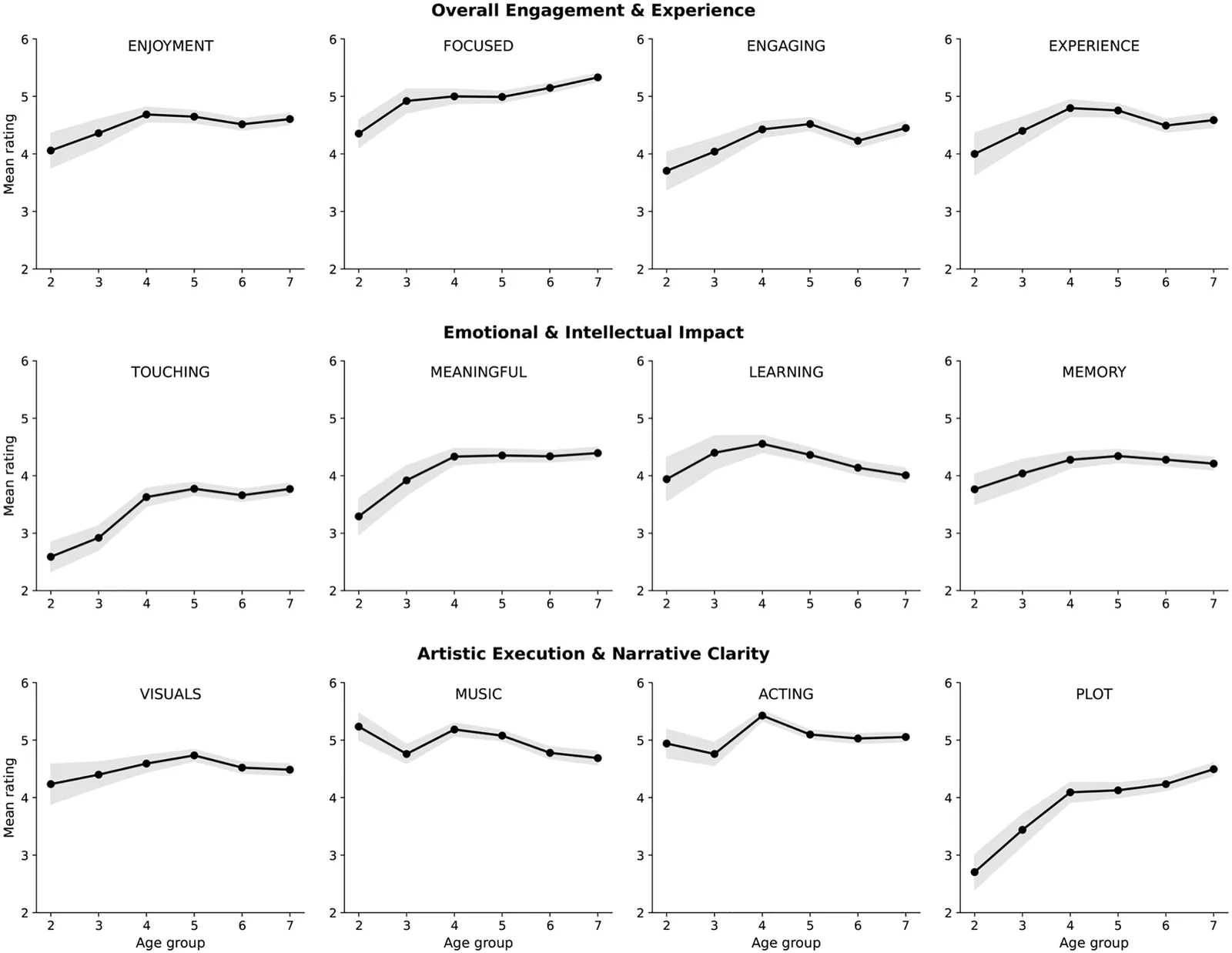
Mean questionnaire ratings across age groups. Age-group codes are: 2 = ≤30 years, 3 = 31–40 years, 4 = 41–50 years, 5 = 51–60 years, 6 = 61–70 years, and 7 = ≥71 years. The original <20-year and 21–30-year categories were combined into group 2, and the 71–80-year and >80-year categories were combined into group 7. Each panel shows the group mean as a black line with circles, with the grey-shaded area indicating ±1 SEM.

To examine relationships and possible redundancy among the questionnaire items, we calculated pairwise Pearson correlations across the 12 items. The correlations show which items elicited similar response patterns and therefore may measure overlapping aspects of audience experience.

The correlation matrix (Fig 11) showed that most inter-item correlations were moderate (0.3–0.6), although several item pairs were strongly correlated (Table 4).

**Fig 11:**
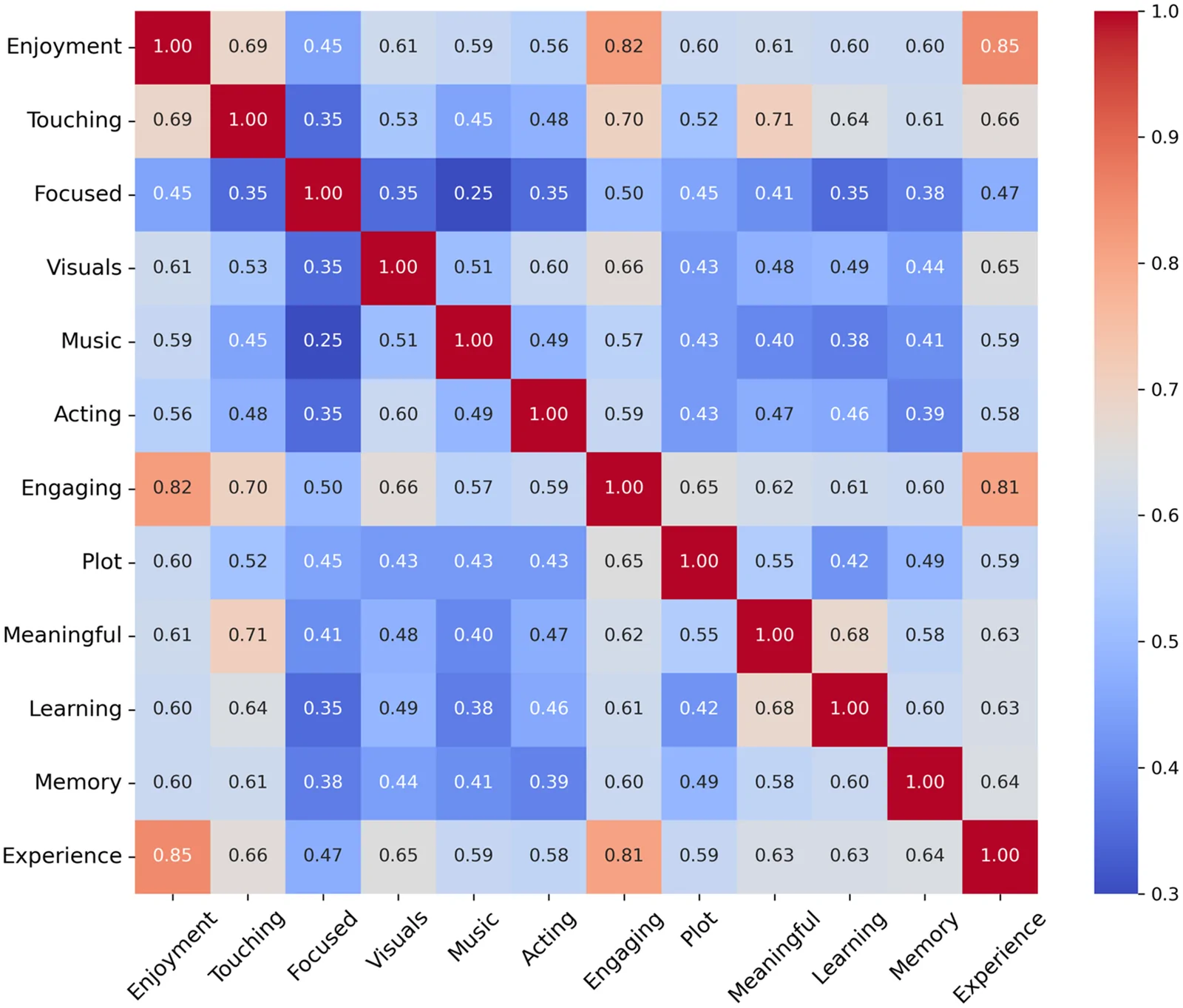
Pairwise Pearson correlations among the 12 questionnaire-item scores.

**Table 4:** Ten highest Pearson correlations among the 12 questionnaire items.

Questionnaire item 1	Questionnaire item 2	*Pearson r*
Experience	Enjoyment	0.85
Engaging	Enjoyment	0.82
Experience	Engaging	0.81
Meaningful	Touching	0.71
Engaging	Touching	0.70
Touching	Enjoyment	0.69
Learning	Meaningful	0.68
Engaging	Visuals	0.66
Experience	Touching	0.66
Plot	Engaging	0.65

### Open-ended responses and sentiment analysis

To complement the cluster analysis, we examined open-ended responses from 268 questionnaire respondents distributed across the three clusters. The open-ended responses provided examples of aspects of audience experience not captured by the numerical ratings. For illustration, we selected the three respondents in each cluster whose standardised 12-item rating profiles were closest to that cluster’s centroid (Fig 9). This provided a transparent quantitative basis for selecting illustrative comments from respondents whose quantitative profiles were closest to the centre of each cluster.

**Audience Cluster 1:**

Male, Age 41–50, Theatre-going frequency 3

“Pretty diluted, interesting and important topics, not very skillfully dealt with. Boring and/or confusing in places. Singing and musical scenes occasionally enlivened. Good role performances, a dull script.”

Female, Age 61–70, Theatre-going frequency 5

“Second half better. Some good role performances. Somewhat confusing overall. Song lyrics partly unclear.”

Male, Age 41–50, Theatre-going frequency 1

“Even after the show, I still don’t know what the plot of the show was. The world creation (zeitgeist) was done well, but the plot arc was unclear.”

**Audience Cluster 2:**

Male, Age 61–70, Theatre-going frequency 5

“First half: good intensity from the whole group. Lottery Girl stole the show, not forgetting Paavo Kerosuo’s brilliant characters. The second half was better for me. The ending was great: a cut to 1981 and a well-written melodic song at the very end. I felt I had seen one of the best plays in the metropolitan area during the autumn season.”

Female, Age 51–60, Theatre-going frequency 5

“All in all, a good performance. I enjoyed. The first half was a bit long. The narration was a bit disjointed. Second half was well condensed. Excellent characters. Kerosuo stretched it out very well. The imagery of childhood/youth came back well and evoked memories/feelings. The video of Kekkonen caused me terrible vicarious embarrassment and at the same time made me happy that things have moved on and developed. Some of the staleness has been removed from society. I felt sorry for Pirkko Työläjärvi (a well-known and appreciated Finnish female politician).”

Female, Age 51–60, Theatre-going frequency 4

“First half: took a while to get into the performance. The second half went by quickly. The performance did not underestimate the audience. It was based on knowledge and experience of the Kekkonen era.”

**Audience Cluster 3:**

Female, Age 51–60, Theatre-going frequency 3

“The performance was pleasantly modern. The characters were caricatured, bringing comedy but also tragedy. The Russian was good and genuinely threatening!”

Female, Age 61–70, Theatre-going frequency 4

“References and images to, e.g., Minna Canth, The Unknown Soldier, Hannu Salama and of course history and pop culture and time. I was left with a long period of reflection. For example, Russia’s attitude to Ukraine or the West now. It was a gripping and impressive performance. Thank you.”

Female, Age 51–60, Theatre-going frequency 4

“The first half: the excellent Lottery Girl, execution memories (involving UKK), Russian appalling pressure, betrayed Sylvi (UKK’s wife). Second half: the desperation of Ahti Karjalainen, the Pauli dance, rainbow trout, Kekkonen’s artistic perversion, fine song performances.”

All 268 open-ended responses were also analysed using VADER to estimate the mean whole-text sentiment in each cluster (Fig 12). Mean VADER compound sentiment scores (range: −1 to +1) were 0.11 ± 0.08 SEM for Cluster 1, 0.38 ± 0.05 SEM for Cluster 2, and 0.52 ± 0.04 SEM for Cluster 3. Mean VADER sentiment increased from Cluster 1 to Cluster 3, paralleling the graded differences observed in the questionnaire ratings.

**Fig 12:**
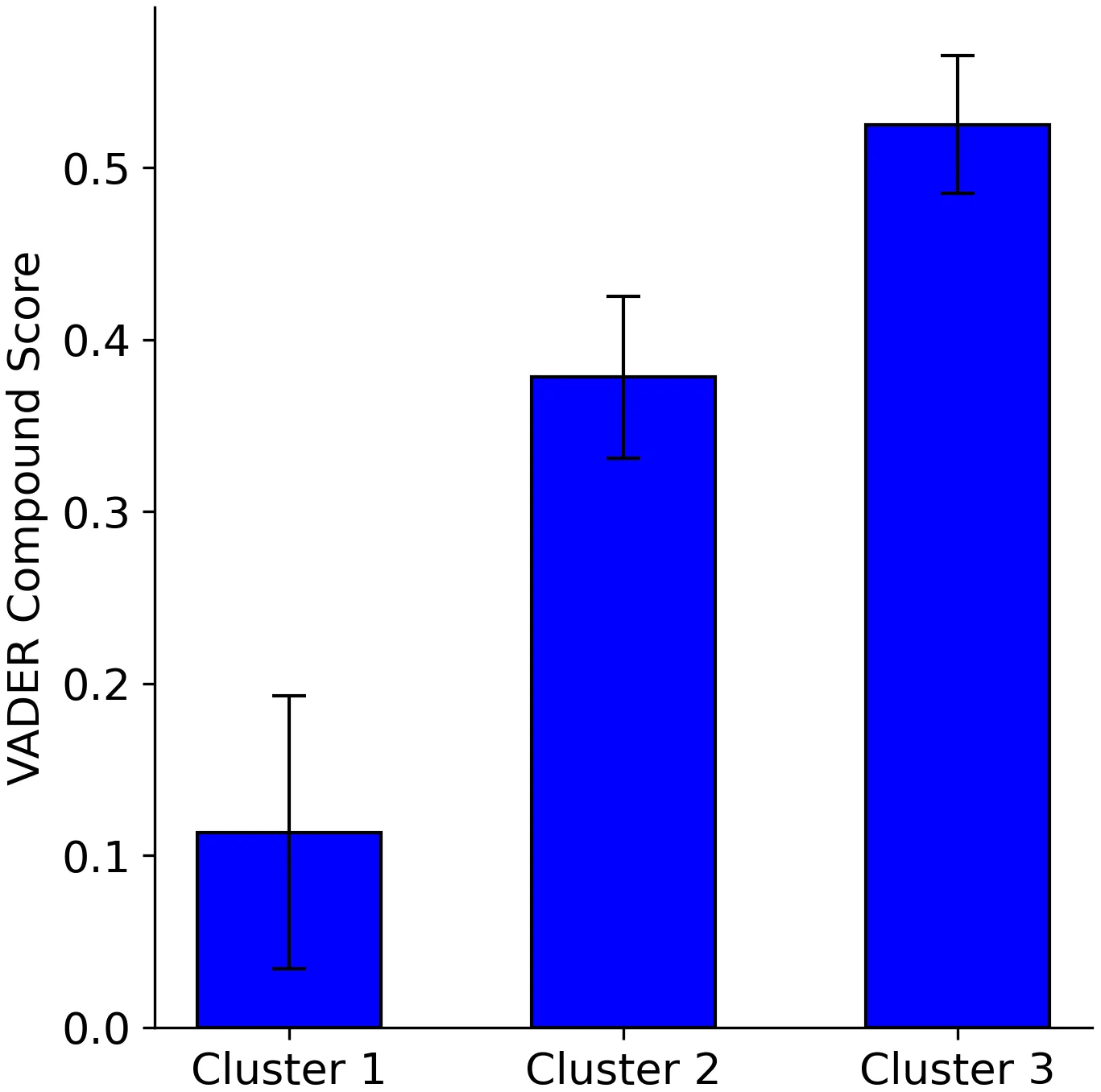
Mean VADER compound sentiment scores by audience cluster. Bars show cluster means, and error bars indicate ±1 SEM. Scores range from −1 (most negative) to +1 (most positive).

Shapiro–Wilk tests indicated non-normal sentiment distributions in all three clusters (Cluster 1: W = 0.944, p = 0.014, n = 54; Cluster 2: W = 0.922, p < 0.001, n = 100; Cluster 3: W = 0.857, p < 0.001, n = 114). Therefore, between-cluster differences in sentiment were evaluated using non-parametric tests. The Kruskal–Wallis test showed that VADER sentiment scores differed significantly among the three clusters (H = 20.046, p < 0.001). Bonferroni-corrected post hoc Mann–Whitney U tests showed significant differences between all cluster pairs (Cluster 1 vs. 2: U = 1976.0, adjusted p = 0.018; Cluster 1 vs. 3: U = 1840.5, adjusted p < 0.001; Cluster 2 vs. 3: U = 4607.0, adjusted p = 0.047).

## Discussion

### Overview of the findings

The study addressed two linked problems: how much audience response varies at the performance and spectator levels when measured in different ways, and how the resulting information may be interpreted and used in theatre practice. Four findings frame the Discussion. First, audience-level enjoyment ratings varied across performances, although the overall reception remained positive. The same production therefore did not generate a fixed response profile. However, the present design cannot determine whether this variation arose from differences in the live performance event, audience composition, or both. Second, kiosk and questionnaire responses to the same enjoyment question did not differ significantly at the aggregate level. The immediate kiosk measure and the more detailed questionnaire thus produced comparable estimates of overall enjoyment while serving different purposes. Third, the questionnaire responses revealed substantial heterogeneity among spectators. The cluster analysis summarised this variation as three graded response profiles, and the open-ended comments illustrated experiences associated with these profiles rather than defining distinct audience types. Fourth, the professional interviews showed that the practical value of audience information depends on what is measured and on how, when, and to whom the findings are communicated. Together, the findings show why a single audience mean or measurement method is insufficient: it can conceal variation between performances, differences among spectators, or practical questions about how the information should be used.

#### Complementary roles of kiosk and questionnaire measures.

The kiosk and questionnaire served different but complementary purposes. The kiosks provided an immediate, low-burden measure of overall enjoyment and produced 1,887 responses, corresponding to 67% of total attendance. The questionnaire required more effort and therefore had lower participation, but it captured several dimensions of experience and allowed individual response profiles to be examined. For the one item shared by both methods, the pooled response distributions did not differ significantly. This finding does not by itself validate either instrument, because the responses could not be linked at the individual level and the questionnaire respondents formed a self-selected subset of the audience. It nevertheless indicates that the two methods did not produce detectably different audience-level estimates of overall enjoyment in this production.

Variation in kiosk ratings across performances is important because it shows that the audience-level response was not a fixed property of the production. However, the repeated-performance design does not isolate the source of this variation. Each showing brought together a distinct live performance event and a different audience, and demographic information was not available for kiosk respondents. The observed differences could therefore reflect variation in the performance, audience composition, interaction between performers and spectators, sampling of respondents, or some combination of these factors. Similarly, the greater variability of kiosk than questionnaire ratings cannot be attributed solely to immediate versus reflective evaluation, because the two methods involved different respondent groups and participation rates. The findings establish that audience-level response profiles differed across showings, but not why they differed.

The exploratory analysis showed a negative association between audience size and mean kiosk enjoyment: smaller audiences tended to give slightly higher ratings. Because only 20 performances were included and audience size was not experimentally manipulated, this association should not be interpreted causally. Audience size may have covaried with the timing of the performance, audience composition, characteristics of the live event, or the likelihood of providing kiosk feedback. It may also influence the social conditions of a performance, including perceived intimacy, collective arousal, and performer–audience interaction, but the present data cannot distinguish among these possibilities. Previous work indicates that audience size can affect physiological arousal and expressive behaviour without producing a simple corresponding change in reported liking [13]. The present finding should therefore be treated as a hypothesis-generating observation about the possible relevance of audience size rather than as an explanation for the variation across performances.

The single-item kiosk design involved a trade-off between breadth and comparability across performances. Asking the same enjoyment question after every showing enabled a low-burden comparison across all 20 performances, but it could not identify which aspects of the production contributed to the ratings. Future studies could retain a common overall-evaluation item while adding or rotating brief questions about plot clarity, acting, music, or emotional impact. The common item would preserve comparability across performances, whereas the additional questions would provide more diagnostic information. Such extensions should remain brief, because the practical value of kiosks depends on minimal interruption and high participation.

### Questionnaire response patterns and audience heterogeneity

The questionnaire complemented the kiosk measure by describing individual audience experiences across 12 dimensions. It was completed by 458 respondents, corresponding to 16% of total attendance. People willing to complete a longer questionnaire may have differed systematically from those who did not respond; therefore, the findings cannot be assumed to represent all spectators. Nevertheless, the questionnaire showed that a generally positive reception coexisted with variation in emotional impact, narrative clarity, and appreciation of specific artistic elements. Its main contribution was therefore not a more accurate estimate of overall enjoyment, but a more differentiated account of how individual spectators evaluated the production.

Although the questionnaire ratings were generally positive, they were not uniformly high across all dimensions. Focused, Acting, and Music received the highest mean ratings, whereas Touching received the lowest. This differentiation is important because it shows that a favourable overall evaluation did not imply equally strong emotional, narrative, and artistic responses. The multidimensional questionnaire therefore revealed distinctions that could not be obtained from the single overall enjoyment item alone.

The PCA provided a descriptive summary of the main patterns of covariation among the questionnaire items. The first component explained most of the variance and was positively associated with enjoyment, engagement, touching, and overall experience. We therefore use the label “Overall Engagement” as a concise description of this general evaluative dimension. The second component explained substantially less variance and was most strongly associated with ratings of music, acting, and visuals; we refer to it as “Production and Artistic Quality.” These labels are interpretive aids rather than validated psychological constructs, and the second component should not be treated as equally important to the dominant general-evaluation dimension.

The three-cluster solution provided a descriptive summary of heterogeneity among questionnaire respondents. Cluster 1 gave the lowest ratings, Cluster 2 intermediate ratings, and Cluster 3 the highest ratings across most questionnaire items. The dominant distinction among the clusters was therefore the general level of evaluation represented by PC1, rather than sharply different combinations of experiential qualities. Differences along PC2 provided some additional separation in the appraisal of music, acting, and visual elements, but this dimension accounted for much less variance. The clusters should not be interpreted as fixed audience types or stable psychological categories. Instead, they summarize graded response profiles within this sample and show that a favourable audience-level mean can conceal substantial variation among individual spectators.

### Open-ended responses and interpretation of audience profiles

The open-ended responses were used to illustrate, rather than independently validate, the quantitative response profiles. Selecting three respondents whose ratings were closest to each cluster centroid linked the comments to characteristic rating patterns, but these nine examples cannot represent the full range of experiences within each cluster. Their value lies in showing how numerical evaluations were expressed through observations about narrative clarity, acting, historical memory, emotional resonance, and contemporary political associations. The comments therefore add interpretive detail to the cluster profiles while remaining illustrative examples rather than a systematic thematic analysis.

The nine selected comments broadly paralleled the graded quantitative response profiles. The Cluster 1 comments combined recognition of individual artistic strengths with criticism of plot clarity, structure, and pacing. The Cluster 2 comments were generally more positive but still mentioned difficulties with the first half, while also connecting the performance with personal memories and Finnish history. The Cluster 3 comments referred to multiple artistic and thematic elements and, in some cases, connected the historical material with contemporary political events. These examples show how differences in overall rating level could be accompanied by differences in what spectators emphasised when describing the performance. However, because only three centroid-nearest respondents from each cluster were selected and the comments were not subjected to systematic thematic analysis, they cannot support broader conclusions about cluster-specific cultural knowledge, identity, or interpretive style.

### Audience demographics and exploratory age associations

Among questionnaire respondents, 64% were women and 78% were 51 years or older. A predominance of women is consistent with theatre-audience surveys in the United States and the United Kingdom [14,15]. The predominance of older respondents is compatible with earlier observations that theatre attendance may be associated with greater disposable income, more flexible leisure time, and cultural capital and theatre-going habits accumulated over the life course [16]. The historical subject matter of UKK may have further contributed to the age profile. Older Finnish spectators were more likely to have direct or family-mediated memories of Kekkonen’s presidency and of the social and political context represented in the production. Such familiarity may have strengthened narrative engagement by connecting the performance with autobiographical memories, national history, and identity [17]. Younger spectators may, by contrast, have had less prior knowledge of Kekkonen and the period, potentially reducing the immediate relevance of some historical and political references. The thematic content of a production may thus contribute to the demographic composition of its audience. These interpretations remain tentative, however, because demographic information was available only from self-selected questionnaire respondents, and we did not measure historical knowledge, income, cultural capital, or reasons for attending.

Within the questionnaire sample, FDR-corrected associations with age group remained for Focused, Touching, Meaningful, Music, and Plot. Mean ratings of Touching, Meaningful, and Plot increased most clearly across the age groups, whereas Music showed a slight decrease with age. The nominal association with Acting did not survive correction. These associations were exploratory and do not establish that ageing itself caused the differences. They may reflect age-related psychological tendencies, greater familiarity with the production’s historical context, generational differences in musical preferences, or differences among the people who chose to complete the questionnaire.

One possible interpretive framework is socioemotional selectivity theory, according to which increasing age is often accompanied by greater emphasis on emotionally meaningful and positively valenced experiences [18]. The related positivity effect may influence attention, memory, and evaluation, potentially contributing to older spectators’ stronger ratings of touching, meaningfulness, and narrative content [18]. Theatre-specific research also suggests age differences in the psychological benefits and evaluative experience of attendance. Meeks and colleagues [15], for example, reported stronger positive affective returns among older theatre-goers. These perspectives are compatible with the present age associations, but they do not fully explain them. In particular, the opposite age trend for Music indicates that age did not simply produce a general tendency to rate every aspect more positively. The findings instead suggest that age may be related differently to emotional, narrative, and artistic dimensions of theatre experience.

### Methodological considerations

A central strength of the study is the collection of audience data across 20 performances of the same production. This repeated-performance design made it possible to examine variation in audience-level responses while recognising that every live performance was a distinct event involving a different audience. The customer-feedback kiosks complemented this design by providing a brief and immediate measure after each performance, with considerably higher participation than the questionnaire. Their principal methodological value was therefore not detailed analysis of individual experience, but consistent low-burden measurement across the full performance series. Because kiosk responses were anonymous and could not be linked to questionnaire responses, however, the design could describe variation across showings but could not determine its source.

Combining kiosk ratings, questionnaire responses, and open-ended comments made it possible to examine variation at complementary levels. The kiosks described overall enjoyment across repeated performances, the questionnaire revealed variation among individual spectators across several dimensions, and the comments illustrated how selected spectators expressed experiences associated with the quantitative profiles. No single method, and no single audience average, captured all these forms of variation. The materials should not be regarded as equivalent or as mutually validating measures. Their combination instead connects broad audience-level patterns with more detailed individual-level accounts. The cluster analysis provided a descriptive way to organise heterogeneity among respondents, while the collaborative development of the questionnaire with theatre practitioners increased its relevance to the production. At the same time, this collaboration produced an instrument designed for the practical context of the study rather than for psychometric optimisation. The methodological contribution therefore lies in measuring different forms of variation under real-world conditions while remaining explicit about the limitations of each method.

The questionnaire was developed collaboratively for the present production rather than as a psychometrically validated scale. Several global evaluation items, particularly Enjoyment, Engaging, and Experience, were strongly correlated and may have captured overlapping aspects of overall engagement or satisfaction. Touching and Meaningful were also closely related. These correlations help explain the dominant general-evaluation dimension identified by the PCA, but they do not by themselves establish reliability, validity, or stable underlying constructs. Future versions of the questionnaire could reduce respondent burden by combining or removing overlapping global items while retaining questions about distinct artistic elements, such as Music, Acting, and Visuals. Formal development of a reusable instrument would additionally require independent psychometric evaluation, including assessment of reliability, construct validity, and replication in other productions and audiences.

Reducing overlap among the generic items would also create space for a small number of production-specific questions. These could be developed with the artistic team to address the themes, dramaturgical choices, or intended effects most relevant to a particular production. Combining a concise common set of items with carefully selected production-specific questions would support comparisons across studies while keeping the measurement connected to the artistic aims of each work.

It would also be valuable to obtain parallel responses from the performers themselves. Their experiential accounts, collected through brief post-show questionnaires or structured debrief interviews, could provide important context for interpreting fluctuations in audience ratings [19]. Performers could rate their perceived energy, focus, pacing, or sense of audience connection after each show. Linking these reports with audience-level data would allow examination of whether performer and audience experiences co-vary across performances. Such associations would not establish causality, but they could help distinguish variation related to the live performance event from variation related to audience composition and clarify the reciprocal character of performer–audience interaction.

### Implications for theatre practice

Audience measurement is useful to theatre practice only if its purpose is clear and its findings can be interpreted without being treated as a definitive judgement of artistic quality. Doing so is not straightforward. Artists and art scholars have noted that empirical approaches may reduce complex artistic and affective experiences to the aspects that are easiest to quantify [20,21]. Quantitative audience data cannot provide a complete account of what a performance means or accomplishes. The interviews with three theatre professionals were included to examine how audience data might be useful to artistic work without becoming a definitive evaluation of it. The interviews were supplementary professional reflections rather than a systematic qualitative study, but they helped place the audience findings within the realities and concerns of theatre practice.

Introducing research procedures into a performance setting may itself influence the event being studied. As Reason [22] observes, “the very act of researching an audience alters its dynamic” (p. 18). The director expressed a related difficulty: “You don’t quite know how you would measure something like audience energy.” The playwright similarly reflected: “It’s really difficult to describe that energy… something happens with the rhythm of the audience that you can feel.” These comments identify an important methodological problem. Theatre professionals experience audience energy and responsiveness as meaningful features of the live event, but these emergent qualities are difficult to operationalise. Kiosk and questionnaire responses provide partial indicators of audience experience; they should not be interpreted as direct measurements of the ongoing relational dynamics between performers and spectators.

The interviews also indicated that the value of audience feedback depends on its timing and purpose. The playwright noted that “real-time feedback isn’t always helpful, especially if it’s not very positive.” The director similarly observed: “Sometimes you don’t even want to know. Or you want to know, but you can’t do anything about it anymore. That’s why maybe it’s better to think of the feedback [as learning material] for the next production, not for the current one.” Once a production is underway, opportunities for substantial revision may be limited, and immediate negative feedback may affect performers without providing a clear basis for action. Audience data may therefore be more useful when collected and communicated for a defined purpose, such as making small adjustments early in a run or informing future productions.

Despite these reservations, all three interviewees regarded some form of audience feedback as potentially useful. The artistic director identified possible applications in small technical adjustments, communication, and future programming. However, the interviewees also stressed that feedback should be developed in communication with the production team and conveyed through an appropriate process. During an ongoing run, for example, results might first be discussed with the producer or the director rather than communicated directly to the cast. The practical value of audience data therefore depends not only on what is measured, but also on when, why, and to whom the findings are presented.

Audience feedback may nevertheless be valuable when the questions are closely aligned with the artistic concerns of the production. The interviewees described how audience reactions—including laughter, silence, applause, and bodily stillness—can influence pacing, delivery, and interpretation during a performance. The playwright proposed reflective questions such as: “Did this play make you think differently about something in life?”, “What good did this play bring to the world?”, and “Did anything about your understanding of empathy shift after seeing this play?” Such questions move beyond general satisfaction and seek to examine the meanings and possible longer-term effects attributed to the performance. Carefully designed audience research could therefore address concerns defined by the artists themselves, while also providing information about audience reach and perceived impact that may be useful to venues, funders, and collaborators.

In sum, audience research is most likely to support theatre practice when its questions, timing, interpretation, and communication are developed with the artistic team. The data should be treated as one source of reflection rather than as a definitive judgement of a production. Used in this way, audience feedback can help artists consider how different spectators respond, identify questions for future productions, and communicate aspects of audience reach and perceived impact without reducing artistic value to numerical ratings.

### Limitations and future research

The questionnaire was developed jointly by academic researchers and theatre professionals, but the time available for co-design was limited. The instrument was therefore tailored to this production rather than developed as a validated general measure of theatre experience. Several items overlapped conceptually, and the questionnaire did not capture every experiential or dramaturgical dimension that might have been relevant to the production. Future studies should allow a longer co-design phase, followed by piloting and psychometric evaluation of the resulting instrument across different productions and audiences.

Demographic information was available only for questionnaire respondents, and kiosk and questionnaire responses could not be linked at the individual level. We therefore cannot determine whether variation in kiosk ratings across performances reflected differences in the live performance event, audience composition, respondent selection, or some combination of these factors. Nor can we assume that the self-selected questionnaire respondents represented the full audience. The findings establish that audience-level enjoyment ratings differed across showings, but they do not identify the causes of that variation.

The present study demonstrates the feasibility of conducting systematic audience research within an ongoing theatre production, but it also shows that methodological and artistic questions cannot be separated completely. Decisions about what to measure, when to collect feedback, and how to interpret and communicate the results affect both the scientific value and the practical usefulness of the research. Future studies should therefore combine stronger methodological planning with continued dialogue among researchers, performers, and other theatre professionals. Such collaboration is needed not only to improve measurement, but also to ensure that audience data are interpreted as partial evidence about a complex live event rather than as a definitive account of its artistic value.

Future studies could complement self-report measures with physiological and behavioural recordings [7], such as heart rate, electrodermal activity, movement, or observable audience responses. Such measures may provide information about the timing and bodily expression of audience reactions, but they do not directly reveal spectators’ subjective experiences. They also require specialised equipment and technical support and may be difficult to implement without affecting the natural performance setting.

## Conclusions

The same theatre production elicited diverse audience responses. Audience-level enjoyment varied across performances, and individual spectators differed substantially in how they experienced the production. The different methods revealed different forms of variation: kiosks supported repeated measurement across showings, while the questionnaire and open-ended responses provided a more detailed account of differences among spectators. Neither a single audience mean nor one measurement method can adequately represent theatre reception.

The feedback from theatre professionals showed that measuring variation is not enough. Audience data become useful to theatre practice only when the questions are relevant to artistic work and the timing, interpretation, and communication of the findings are considered carefully. Systematic audience research can therefore contribute to theatre, but its results remain partial, context-dependent, and insufficient for judging artistic quality. Audience research can reveal patterns in spectators’ responses and help clarify how performance-specific factors, such as audience size and responsiveness, variation in acting, and other differences between showings, relate to overall satisfaction. These findings can support informed reflection and dialogue between researchers and theatre professionals and help integrate research more closely into theatre-making and other creative processes.

## Supporting information

S1 Appendix Original Finnish version of the UKK music theatre audience questionnaire. (PDF)

S2 Appendix English translation of the UKK music theatre audience questionnaire. (PDF)
