# Supplementary material for: Measuring audience experience across 20 showings of the same theatre performance using customer feedback kiosks and a questionnaire
Source: PLoS One. 2026 Oct 22;21(10):e0344225. doi: 10.1371/journal.pone.0344225 (PMC13690226; doi:10.1371/journal.pone.0344225)
Supplement: Supplementary file 1 [file pone.0344225.s001.pdf]

# UKK-musiikkinäytelmän katsojatutkimus

Pakolliset kysymykset merkitty tähdellä (*)

Kiitos, että otat hetken osallistuaksesi tähän Aalto-yliopiston, Greta Tuotannon ja Musiikkiteatteri Kapsäkin yhteiseen tutkimukseen. Pyydämme sinua palauttamaan mieleesi UKK-musiikkinäytelmän, jonka näit Kapsäkissä. Tämä kysely on helppo täyttää joko kännykällä tai tietokoneella, missä tahansa sinulle parhaiten sopii.

Vastaa jokaiseen kysymykseen rauhassa ja luottaen omaan intuitioosi – oikeita tai vääriä vastauksia ei ole. Olethan mahdollisimman rehellinen, sillä vastauksesi auttavat meitä ymmärtämään esityksen vaikutuksia katsojiin ja antamaan arvokasta palautetta esityksen tekijöille. Kysymykset jakautuvat neljälle näytölle.

Kiitos osallistumisestasi ja arvokkaasta palautteestasi! Arvomme kyselyyn osallistujien kesken vapaalippuja Musiikkiteatteri Kapsäkin ja Greta tuotannon esityksiin. Kyselyyn vastanneiden yhteystietoja ei tallenneta automaattisesti. Annathan tämän kyselyn lopuksi yhteystietosi erillisellä lomakkeella, mikäli haluat osallistua arvontaan. Osallistuttuasi arvontaan saat vielä mahdollisuuden tilata Greta Tuotannon uutiskirjeen.

## 1. Kuinka paljon nautit esityksestä (1 = en lainkaan, 6 = erittäin paljon)?

| 1 | 2 | 3 | 4 | 5 | 6 |
|---|---|---|---|---|---|
| ○ | ○ | ○ | ○ | ○ | ○ |

## 2. Kuinka syvästi esitys kosketti sinua? (1= ei koskettanut lainkaan, 6 = erittäin syvästi)?

| 1 | 2 | 3 | 4 | 5 | 6 |
|---|---|---|---|---|---|
| ○ | ○ | ○ | ○ | ○ | ○ |

3. Kuinka tarkkaavainen olit esityksen aikana (1 = ajattelin muita asioita, 6 = seurasin esitystä hyvin intensiivisesti)?

| | 1 | 2 | 3 | 4 | 5 | 6 |
|---|---|---|---|---|---|---|
| | ○ | ○ | ○ | ○ | ○ | ○ |

4. Kuinka onnistuneeksi koit esityksen tunnelman luomisessa sen visuaalisuuden; lavastuksen, puvut ja valaistuksen (1 = en lainkaan onnistuneeksi, 6 = erittäin onnistuneeksi)?

| | 1 | 2 | 3 | 4 | 5 | 6 |
|---|---|---|---|---|---|---|
| | ○ | ○ | ○ | ○ | ○ | ○ |

5. Kuinka onnistuneeksi koit musiikin ja äänimaiseman tunnelman luomisessa (1 = en lainkaan onnistuneeksi, 6 = erittäin onnistuneeksi)?

| | 1 | 2 | 3 | 4 | 5 | 6 |
|---|---|---|---|---|---|---|
| | ○ | ○ | ○ | ○ | ○ | ○ |

6. Miten arvioisit näyttelijöiden roolisuoritukset (1= eivät onnistuneet lainkaan, 6 = olivat erinomaisia)?

| | 1 | 2 | 3 | 4 | 5 | 6 |
|---|---|---|---|---|---|---|
| | ○ | ○ | ○ | ○ | ○ | ○ |

7. Kuinka mukaansatempaavaksi koit esityksen (1 = en lainkaan mukaansatempaavaksi, 6 = erittäin mukaansatempaavaksi)?

| | 1 | 2 | 3 | 4 | 5 | 6 |
|---|---|---|---|---|---|---|
| | ○ | ○ | ○ | ○ | ○ | ○ |

8. Kuinka selkeänä pidit tarinaa ja juonen kulkua (1 = oli vaikea seurata, 6 = oli erittäin helppo seurata)?

| 1 | 2 | 3 | 4 | 5 | 6 |
|---|---|---|---|---|---|
| ○ | ○ | ○ | ○ | ○ | ○ |

**9. Kuinka merkityksellisiksi tai samastuttaviksi koit näytelmän teemat (1 = teemat eivät koskettaneet minua lainkaan, 6 = koin teemat erittäin merkityksellisinä)**

| 1 | 2 | 3 | 4 | 5 | 6 |
|---|---|---|---|---|---|
| ○ | ○ | ○ | ○ | ○ | ○ |

**10. Saitko esityksestä ajateltavaa tai pohdittavaa (1 = en lainkaan, 6 = näytelmä avasi minulle valtavasti uusia näkökulmia ja pohdittavaa)?**

| 1 | 2 | 3 | 4 | 5 | 6 |
|---|---|---|---|---|---|
| ○ | ○ | ○ | ○ | ○ | ○ |

**11. Kuinka pitkään arvioit muistavasi esityksen (1 = unohdan nopeasti, 6 = esitys säilyy pitkään mielessäni)?**

| 1 | 2 | 3 | 4 | 5 | 6 |
|---|---|---|---|---|---|
| ○ | ○ | ○ | ○ | ○ | ○ |

**12. Kuinka tyytyväinen olet teatterikokemukseen kokonaisuudessaan (1 = olisin voinut yhtä hyvin jättää menemättä, 6 = nautin koko esityksestä täysin siemauksin)?**

| 1 | 2 | 3 | 4 | 5 | 6 |
|---|---|---|---|---|---|
| ○ | ○ | ○ | ○ | ○ | ○ |

**13. Kuinka todennäköisesti suosittelisit tätä esitystä muille (1 = en voi suositella lainkaan, 10 = suosittelen näytelmää kaikille tapaamilleni ihmisille todella lämpimästi)?**

| 1 | 2 | 3 | 4 | 5 | 6 | 7 | 8 | 9 | 10 |
|---|---|---|---|---|---|---|---|---|---|
| ○ | ○ | ○ | ○ | ○ | ○ | ○ | ○ | ○ | ○ |

**14. Voit kirjoittaa haluamassasi laajuudessa mitä tahansa, mitä sinulle jäi mieleen esityksestä. Jäikö mieleesi jotain erityistä? Käytä tässä mallia: 1) Ensimmäinen puoliaika, 2) Toinen puoliaika.**

**15. Vastaajan sukupuoli ***

○ Mies ○ Nainen ○ Muu ○ En halua kertoa

**16. Vastaajan ikä ***

○ alle 20 ○ 21-30 ○ 31-40 ○ 41-50 ○ 51-60 ○ 61-70
○ 71-80 ○ yli 80

**17. Käyn teatterissa erittäin harvoin (1) – hyvin usein (6) ***

| 1 | 2 | 3 | 4 | 5 | 6 |
|---|---|---|---|---|---|
| ○ | ○ | ○ | ○ | ○ | ○ |

**18. Olen osallistunut esitykseen**

○ Katsojana

○ Esiintyjänä

- ◯ Taiteellisena suunnittelijana
- ◯ Teknisenä henkilönä
- ◯ Muussa roolissa

### 19. Päivämäärä, jolloin olit katsomassa esityksen *

pp.kk.vvvv

### 20. Esityksen alkamisaika *

### 21. Haluatko osallistua teatterilippujen arvontaan ja/tai osallistua ryhmähaastatteluun, jossa keskustelemme yleisesti taidekokemuksista (ei UKK -esityksestä)? Kyselyyn vastanneiden yhteystietoja ei tallenneta automaattisesti. Vain Kyllä -vastaus tähän kysymykseen johtaa erilliselle lomakkeelle, jolla kysymme lupaa tallentaa yhteystietosi. *

- ◯ Kyllä
- ◯ En
