# Supplementary material for: Measuring audience experience across 20 showings of the same theatre performance using customer feedback kiosks and a questionnaire
Source: PLoS One. 2026 Oct 22;21(10):e0344225. doi: 10.1371/journal.pone.0344225 (PMC13690226; doi:10.1371/journal.pone.0344225)
Supplement: Supplementary file 2 [file pone.0344225.s002.pdf]

UKK Music Theatre Audience Survey

Required questions are marked with an asterisk (*)

Thank you for taking a moment to participate in this joint study by Aalto University, Greta Tuotanto, and Music Theatre Kapsäkki. We ask you to recall the UKK music theatre performance you saw at Kapsäkki. This survey is easy to complete on either a mobile phone or a computer, wherever is most convenient for you.

Please answer each question at your own pace and trust your intuition - there are no right or wrong answers. Please be as honest as possible, as your responses will help us understand the effects of the performance on audience members and provide valuable feedback to the production team. The questions are divided across four screens.

Thank you for participating and for your valuable feedback! Free tickets to performances by Music Theatre Kapsäkki and Greta Tuotanto will be raffled among survey participants. Contact details of questionnaire respondents are not recorded automatically. At the end of this survey, please provide your contact details on a separate form if you wish to enter the draw. After entering the draw, you will also have an opportunity to subscribe to the Greta Tuotanto newsletter.

1. How much did you enjoy the performance? (1 = not at all, 6 = very much)

	1	2	3	4	5	6
	○	○	○	○	○	○

2. How deeply did the performance move you? (1 = not at all, 6 = very deeply)

	1	2	3	4	5	6
	○	○	○	○	○	○

3. How attentive were you during the performance? (1 = I was thinking about other things, 6 = I followed the performance very intensively)

1	2	3	4	5	6
○	○	○	○	○	○

4. How successful did you find the creation of atmosphere through the visuals (set design, costumes, lighting)? (1 = not at all successful, 6 = very successful)

1	2	3	4	5	6
○	○	○	○	○	○

5. How successful did you find the creation of atmosphere through the music and soundscape? (1 = not at all successful, 6 = very successful)

1	2	3	4	5	6
○	○	○	○	○	○

6. How would you evaluate the actors' performances? (1 = not at all successful, 6 = excellent)

1	2	3	4	5	6
○	○	○	○	○	○

7. How engaging did you find the performance? (1 = not at all engaging, 6 = very engaging)

1	2	3	4	5	6
○	○	○	○	○	○

8. How clear did you find the story and narrative progression? (1 = very difficult to follow, 6 = very easy to follow)

	1	2	3	4	5	6
	○	○	○	○	○	○

9. How meaningful or relatable did you find the themes of the play? (1 = the themes did not move me at all, 6 = I found the themes very meaningful)

	1	2	3	4	5	6
	○	○	○	○	○	○

10. Did the performance give you something to think about or reflect on? (1 = not at all, 6 = the play gave me a great many new perspectives and things to reflect on)

	1	2	3	4	5	6
	○	○	○	○	○	○

11. How long do you expect to remember the performance? (1 = I will forget it quickly, 6 = the performance will remain in my mind for a long time)

	1	2	3	4	5	6
	○	○	○	○	○	○

12. How satisfied are you with the overall theatre experience? (1 = I might just as well not have gone, 6 = I thoroughly enjoyed the entire performance)

	1	2	3	4	5	6
	○	○	○	○	○	○

13. How likely are you to recommend this performance to others? (1 = I cannot recommend it at all, 10 = I would recommend the play very warmly to everyone I meet)

1	2	3	4	5	6	7	8	9	10
○	○	○	○	○	○	○	○	○	○

14. You may write, at whatever length you wish, about anything that remained in your mind from the performance. Was there anything in particular that stayed with you? Please use the following structure: 1) First half, 2) Second half.

15. Respondent's gender *

○ Man ○ Woman ○ Other ○ Prefer not to say

16. Respondent's age *

○ under 20 ○ 21-30 ○ 31-40 ○ 41-50 ○ 51-60 ○ 61-70
○ 71-80 ○ over 80

17. I attend the theatre very rarely (1) - very often (6) *

1	2	3	4	5	6
○	○	○	○	○	○

18. I participated in the performance as

○ an audience member

○ a performer

- an artistic designer
- a member of the technical staff
- another role

19. Date on which you saw the performance *

dd.mm.yyyy

20. Performance start time *

21. Would you like to enter the theatre-ticket draw and/or take part in a group interview in which we discuss art experiences in general (not the UKK performance)? Contact details of questionnaire respondents are not recorded automatically. Only a 'Yes' response to this question leads to a separate form on which we ask for permission to record your contact details. *

- Yes
- No
