# Supplementary material for: Measuring audience experience across 20 showings of the same theatre performance using customer feedback kiosks and a questionnaire
Source: PLoS One. 2026 Oct 22;21(10):e0344225. doi: 10.1371/journal.pone.0344225 (PMC13690226; doi:10.1371/journal.pone.0344225)
Supplement: Supplementary file 3 [file pone.0344225.s003.docx]

# Response to the Academic Editor and Reviewers

*Manuscript: "Measuring audience experience across 20 showings of the same theatre performance using customer feedback kiosks and a questionnaire" (PONE-D-25-66243)*

Dear Academic Editor,

Thank you for the opportunity to revise our manuscript and for the careful and constructive reviews. We believe that the MS has improved sustantially after revisions. We have revised the manuscript extensively while retaining its overall structure. The principal changes are: (1) a clearer overarching research question and hierarchy of analyses; (2) an operational definition of collective audience experience; (3) fuller reporting and justification of the kiosk, questionnaire, clustering, demographic, and sentiment-analysis procedures; (4) a substantially reorganised Discussion integrating the different forms of evidence; (5) more cautious interpretation of variation across performances and of the cluster solution; (6) transparent presentation of the complete questionnaire in Finnish and English as Supporting Information; and (7) improved figures, tables, captions, and statistical reporting.

We hope that these revisions satisfactorily address the concerns raised by the Academic Editor and reviewers, and that the manuscript will now be considered suitable for publication in PLOS ONE.

## Major substantive revisions are highlighted in yellow in the revised manuscript. The manuscript has been substantially reorganised in response to the reviewers’ comments, including changes to the structure and integration of the Introduction, Methods, Results, and Discussion. These structural changes may not be fully apparent from the highlighting alone. Page and line references below refer to the revised manuscript.

## Academic Editor

**Comment:** *Major revision was requested on the basis of the reviewer comments.*

**Response:** We have addressed the comments point by point below. The revision clarifies the study's focus, strengthens methodological reporting, moderates claims that extended beyond the data, and develops the methodological and practical implications of combining kiosk ratings, questionnaire data, open-ended responses, and professional perspectives.

**Comment:** *Journal requirements: please provide additional detail on informed consent, ensure the ethics statement appears only in Methods, finalise the data availability statement, and ensure each author is linked to an affiliation.*

**Response:** The Methods section now describes the consent procedures for both the audience data collection and the later professional interviews in full (see response to Reviewer 1 and comment 2.5 above). The ethics statement, including approval details and the consent procedure for the professional interviews, appears only in the Methods section. Author affiliations have been reviewed and corrected. The data availability statement has been finalised in the submission system.

**Location in revised manuscript:** Ethics statement, Methods, p. 14, l. 301–310; interview consent, p. 9, l. 214–216.

## Reviewer 1

**Comment:** *The manuscript was evaluated positively. Three minor corrections were requested: “memebers” → “members” (line 575); author name “Ruotoistenäki” → “Ruotoistenmäki”; and clarification of the informed consent procedure and questionnaire administration method in Methods.*

**Response:** We thank the reviewer for the positive assessment. The spelling error and author name have been corrected throughout. The Methods section now clarifies how spectators were informed about the study and invited to participate via the kiosks and questionnaire.

**Location in revised manuscript:** Methods (Data collection), p. 7, l. 138–141.

## Reviewer 2

**Comment:** *2.1. The manuscript lacks a clear research question and therefore appears unfocused.*

**Response:** We agree that the original manuscript did not state the central research question clearly enough. We reformulated the end of the Introduction to state one overarching question — how immediate kiosk ratings and more detailed post-performance questionnaire data can be combined to characterise audience-level response patterns across repeated showings of the same production — and identify three principal analyses that address it, distinguishing them from the audience-size and demographic analyses, which are now explicitly framed as exploratory.

**Location in revised manuscript:** Introduction, p. 5, l. 97–103.

**Comment:** *2.2. The kiosk, questionnaire, qualitative material, and interviews are presented too separately; their relationship is unclear.*

**Response:** We revised the Introduction and Discussion to distinguish and integrate the roles of the four materials: kiosks provide an immediate, low-burden measure of overall enjoyment; the questionnaire provides multidimensional individual response profiles; the open-ended comments illustrate experiences associated with those profiles; and the interviews examine how audience information might be interpreted and communicated in theatre practice. The revised Discussion now opens with an integrated overview of the four linked findings before developing these roles further.

**Location in revised manuscript:** Introduction, p. 6, l. 105–113; Discussion overview, p. 28, l. 582–585.

**Comment:** *2.3. The statistical analysis is rigorous but too broad and insufficiently directed by a research question.*

**Response:** We now identify variation across performances, the kiosk–questionnaire comparison, and questionnaire response profiles as the three principal analyses (see response to 2.1), while explicitly describing the audience-size and demographic analyses as exploratory. For the demographic analyses, we applied Benjamini–Hochberg false-discovery-rate correction within each demographic factor and revised the Results and interpretation accordingly.

**Location in revised manuscript:** Data analysis, p. 12, l. 270–272; Results, p. 21, l. 453–456.

**Comment:** *2.4. The questionnaire questions are not fully or clearly included.*

**Response:** Figure 1 now presents the 12 scaled items that formed the principal quantitative variables. In addition, the complete questionnaire as administered is supplied as S1 Appendix (original Finnish) and S2 Appendix (English translation), preserving the original order, response alternatives, and item structure.

**Location in revised manuscript:** Fig 1 and caption, p. 8, l. 156–160, 166–167; Methods, p. 9, l. 187–188; S1 and S2 Appendices.

**Comment:** *2.5. The kiosk collection machine and procedure need clearer description.*

**Response:** We expanded the Methods to report the kiosk screen question and response scale, the locations of all three kiosks, anonymous storage of each button press, the absence of personal or demographic data collection, the inability to link kiosk and questionnaire responses, the lack of a technical restriction on repeated presses, and the provider of the kiosk hardware and data system.

**Location in revised manuscript:** Methods (Data collection), p. 7, l. 135–147.

**Comment:** *2.6. The number of kiosk participants was stated too definitively because the system did not identify unique respondents.*

**Response:** We changed the wording throughout from a number of kiosk participants to a number of recorded responses, since the kiosks could not identify unique respondents. The Methods now state explicitly that repeated presses by the same person were not technically prevented and therefore cannot be ruled out (see response to 2.5).

**Location in revised manuscript:** Abstract, p. 3, l. 37–38; Results, p. 15, l. 328.

**Comment:** *2.7. Table 1 contains unclear symbols, and some figures or appendix material are difficult to follow.*

**Response:** We revised the presentation throughout. Table 1 now explains its em-dash notation in a clarifying note. Figure 9 identifies the cluster colours in its caption. Figure 10 explains the age-group codes and the merged extreme categories. Table 3 reports both raw and FDR-adjusted p values (see response to 2.3).

**Location in revised manuscript:** Table 1 note, p. 17, l. 374–375; Fig 9 caption, p. 19, l. 423–425; Fig 10 caption, p. 23, l. 473–477.

**Comment:** *2.8. The Methods section should give more justification for the chosen methods, and methodological considerations should not appear only late in the paper.*

**Response:** We strengthened the rationale for combining immediate kiosk feedback with a multidimensional questionnaire at the start of the Introduction, explaining why no single method captures both between-performance and between-spectator variation. The Discussion retains a separate “Methodological considerations” section, but this is now reserved for broader implications rather than information needed to understand the analyses, which has been moved earlier into Methods.

**Location in revised manuscript:** Introduction, p. 5, l. 83–92.

**Comment:** *2.9. Applause and CO₂ measurements should not be mentioned if they are not analysed in this article.*

**Response:** We removed the references to applause counts and CO₂ monitoring from both Methods and Discussion, since these measures are not analysed in the present article.

**Location in revised manuscript:** Deletion only.

**Comment:** *2.10. The manuscript needs greater academic depth and contextual interpretation.*

**Response:** We expanded the Discussion substantially. It now integrates the principal findings, distinguishes what the design can and cannot establish, develops the complementary roles and limitations of the two measurement approaches (see response to 2.2), interprets the cluster solution cautiously (see response to 3.4), discusses questionnaire overlap (see response to 3.5), and uses the professional interviews to examine the timing, purpose, and communication of audience feedback in theatre practice, situating this within the scholarly literature on empirical approaches to artistic experience.

**Location in revised manuscript:** Discussion, “Implications for theatre practice,” p. 37, l. 798–807.

## Reviewer 3

**Comment:** *3.1. The term “identical performances” is conceptually misleading.*

**Response:** We agree. We removed claims that the performances were identical. The revised manuscript consistently refers to repeated live performances of the same production and recognises that each showing was a distinct event, even though the production itself remained largely unchanged.

**Location in revised manuscript:** Abstract, p. 3, l. 32–34; Introduction, p. 4, l. 57–59.

**Comment:** *3.2. Variation across performances may reflect audience composition as well as properties of the live event.*

**Response:** We now state explicitly that the design cannot determine whether differences arose from the live performance event, audience composition, respondent selection, or their interaction. Demographic information was unavailable for kiosk respondents, and kiosk and questionnaire responses could not be linked. The audience-size association is described as exploratory and hypothesis-generating rather than causal.

**Location in revised manuscript:** Discussion (Limitations), p. 39–40, l. 867–871.

**Comment:** *3.3. “Collective audience experience” needs a clearer theoretical and operational definition.*

**Response:** We added an operational definition. In this study, collective audience experience refers to the distribution and central tendency of individual responses within a performance event. It does not imply that all spectators shared the same subjective experience; rather, each performance produced an audience-level response profile that can be compared across repeated showings.

**Location in revised manuscript:** Introduction, p. 4, l. 68–72.

**Comment:** *3.4. Cluster interpretation should be more cautious and elaborated.*

**Response:** We revised both Results and Discussion to describe the clusters as graded response profiles rather than fixed audience types. We explain that the dominant distinction was the general level of evaluation, that the second component accounted for substantially less variance, and that the nine selected comments are illustrative rather than a systematic qualitative analysis. Claims about stable psychological categories have been removed.

**Location in revised manuscript:** Discussion, p. 32, l. 676–679.

**Comment:** *3.5. Some questionnaire items overlap conceptually.*

**Response:** We now discuss the strong correlations among Enjoyment, Engaging, and Experience and between Touching and Meaningful. We state that these items may capture overlapping aspects of evaluation, that the present instrument was developed for the practical context rather than psychometric optimisation, and that future versions could reduce respondent burden by combining or removing overlapping items while retaining distinct artistic dimensions.

**Location in revised manuscript:** Discussion, p. 35, l. 768–775.
