# Supplementary material for: Measuring audience experience across 20 showings of the same theatre performance using customer feedback kiosks and a questionnaire
Source: PLoS One. 2026 Oct 22;21(10):e0344225. doi: 10.1371/journal.pone.0344225 (PMC13690226; doi:10.1371/journal.pone.0344225)
Supplement: Peer review history

# Peer review: Decision Letter 0

**DOI:** 10.1371/journal.pone.0344225.r001

**Authors:** Manuel Herrador

4 May 2026

PLOS One

Dear Dr. Sams,

Thank you for submitting your manuscript to PLOS ONE. After careful consideration, we feel that it has merit but does not fully meet PLOS ONE’s publication criteria as it currently stands. Therefore, we invite you to submit a revised version of the manuscript that addresses the points raised during the review process.

===================================

Dear authors,

Given the comments received by the reviewers, we recommend a major revision of your work. Please provide it so we can proceed with the next round.

Thanks for your contribution to PLOS ONE

===================================

Please submit your revised manuscript by Jun 18 2026 11:59PM. If you will need more time than this to complete your revisions, please reply to this message or contact the journal office at plosone@plos.org. When you're ready to submit your revision, log on to https://www.editorialmanager.com/pone/ and select the 'Submissions Needing Revision' folder to locate your manuscript file.

Please include the following items when submitting your revised manuscript:

- A letter that responds to each point raised by the academic editor and reviewer(s). You should upload this letter as a separate file labeled 'Response to Reviewers'.
- A marked-up copy of your manuscript that highlights changes made to the original version. You should upload this as a separate file labeled 'Revised Manuscript with Track Changes'.
- An unmarked version of your revised paper without tracked changes. You should upload this as a separate file labeled 'Manuscript'.

If you would like to make changes to your financial disclosure, please include your updated statement in your cover letter. Guidelines for resubmitting your figure files are available below the reviewer comments at the end of this letter.

If applicable, we recommend that you deposit your laboratory protocols in protocols.io to enhance the reproducibility of your results. Protocols.io assigns your protocol its own identifier (DOI) so that it can be cited independently in the future. For instructions see: https://journals.plos.org/plosone/s/submission-guidelines#loc-laboratory-protocols. Additionally, PLOS ONE offers an option for publishing peer-reviewed Lab Protocol articles, which describe protocols hosted on protocols.io. Read more information on sharing protocols at https://plos.org/protocols?utm_medium=editorial-email&utm_source=authorletters&utm_campaign=protocols.

As the corresponding author, your ORCID iD is verified in the submission system and will appear in the published article. PLOS supports the use of ORCID, and we encourage all coauthors to register for an ORCID iD and use it as well. Please encourage your coauthors to verify their ORCID iD within the submission system before final acceptance, as unverified ORCID iDs will not appear in the published article. *Only*  the individual author can complete the verification step; PLOS staff *cannot*  verify ORCID iDs on behalf of authors.

We look forward to receiving your revised manuscript.

Kind regards,

Manuel Herrador, Ph.D.

Academic Editor

PLOS One

Journal Requirements:

When submitting your revision, we need you to address these additional requirements.

1. Please ensure that your manuscript meets PLOS ONE's style requirements, including those for file naming. The PLOS ONE style templates can be found at

https://journals.plos.org/plosone/s/file?id=wjVg/PLOSOne_formatting_sample_main_body.pdf and https://journals.plos.org/plosone/s/file?id=ba62/PLOSOne_formatting_sample_title_authors_affiliations.pdf

2. Please provide additional details regarding participant consent. In the ethics statement in the Methods and online submission information, please ensure that you have specified (1) whether consent was informed and (2) what type you obtained (for instance, written or verbal, and if verbal, how it was documented and witnessed). If your study included minors, state whether you obtained consent from parents or guardians. If the need for consent was waived by the ethics committee, please include this information.

If you are reporting a retrospective study of medical records or archived samples, please ensure that you have discussed whether all data were fully anonymized before you accessed them and/or whether the IRB or ethics committee waived the requirement for informed consent. If patients provided informed written consent to have data from their medical records used in research, please include this information.

3. Your ethics statement should only appear in the Methods section of your manuscript. If your ethics statement is written in any section besides the Methods, please delete it from any other section.

4. When completing the data availability statement of the submission form, you indicated that you will make your data available on acceptance. We strongly recommend all authors decide on a data sharing plan before acceptance, as the process can be lengthy and hold up publication timelines. Please note that, though access restrictions are acceptable now, your entire data will need to be made freely accessible if your manuscript is accepted for publication. This policy applies to all data except where public deposition would breach compliance with the protocol approved by your research ethics board. If you are unable to adhere to our open data policy, please kindly revise your statement to explain your reasoning and we will seek the editor's input on an exemption. Please be assured that, once you have provided your new statement, the assessment of your exemption will not hold up the peer review process.

5. Please amend your list of authors on the manuscript to ensure that each author is linked to an affiliation. Authors’ affiliations should reflect the institution where the work was done (if authors moved subsequently, you can also list the new affiliation stating “current affiliation:….” as necessary).

6. If the reviewer comments include a recommendation to cite specific previously published works, please review and evaluate these publications to determine whether they are relevant and should be cited. There is no requirement to cite these works unless the editor has indicated otherwise.

Additional Editor Comments :

Dear authors,

Given the comments received by the reviewers, we recommend a major revision of your work. Please provide it so we can proceed with the next round.

Thanks for your contribution to PLOS ONE

[Note: HTML markup is below. Please do not edit.]

Reviewers' comments:

Reviewer's Responses to Questions

**Comments to the Author**

1. Is the manuscript technically sound, and do the data support the conclusions?

Reviewer #1: Yes

Reviewer #2: Partly

Reviewer #3: Partly

**********

2. Has the statistical analysis been performed appropriately and rigorously?

Reviewer #1: Yes

Reviewer #2: Yes

Reviewer #3: Yes

**********

3. Have the authors made all data underlying the findings in their manuscript fully available?

The PLOS Data policy

Reviewer #1: Yes

Reviewer #2: Yes

Reviewer #3: Yes

**********

4. Is the manuscript presented in an intelligible fashion and written in standard English?

Reviewer #1: Yes

Reviewer #2: Yes

Reviewer #3: Yes

**********

Reviewer #1: This is a well-executed study with genuine originality and solid technical rigor. The design across 20 identical performances is rare and effectively isolates performance-specific variations from audience characteristics. The mixed-methods approach combining immediate kiosk feedback with reflective questionnaires and qualitative analysis is methodologically sound, with validation showing kiosk and questionnaire responses to identical questions produce equivalent distributions (Mann-Whitney U, p=0.687).

Statistical analysis is appropriate throughout. Chi-square tests correctly assess distribution differences across shows, Type II ANOVA handles unequal demographic groups, and non-parametric Kruskal-Wallis testing appropriately addresses violated normality assumptions in sentiment analysis. Effect sizes are reported and practical significance contextualized—the audience size correlation (R²=0.248) is rightly noted as statistically significant but small in practical terms.

Data transparency is exemplary. Complete dataset deposited at Zenodo with persistent DOI enables full independent verification of findings. The ethical approach is particularly commendable, with genuine engagement of artist perspectives on measurement tensions rather than imposing external frameworks.

Three minor corrections needed: “memebers” to “members” (line 575); author name “Ruotoistenäki” to “Ruotoistenmäki”. Clarify informed consent procedure and questionnaire administration method in Methods section. These are standard copyediting issues not affecting manuscript substance.

Reviewer #2: This article reports the findings from data collected and analysed from 20 shows, it contains rich audience data and tries to demonstrate the importance of “scientific audience data reflection” in theatre artistic creation. There are extensive quantitative data analysis which shows high potential of the research. However, this article lacks a clear research question which leads to a rather focus-less analysis of the data. The results from the data analysis are well described but the more meaningful part of data analysis is missing: how to interpret the statistical analysis, is significantly missing. And I think a clear research question would help the authors to re-structure the article and emphasize the findings and the contributions in theoretical and practical better.

And the other comments are followed:

1. Is the manuscript technically sound, and do the data support the conclusions?

The manuscript demonstrates an interesting research project with extensive data analysis process. In the “Methods” and “Results” sections, the authors explained the technical process in detail. However, the data collection and analysis lacks a clear direction: there is no research question asked in this research project. Consequently, the whole article is presented in a fragmented manner, and the results from data analysis failed to go deeper in either theoretical nor practical perspective. For instance, the purpose of the data collection design which including kiosk responds, questionnaire and the onsite audience interviews is not clear, if it’s essential to include the data from all three perspectives, I would expect to read the interactions of the findings from the three perspectives instead of simply description of them individually.

2. Has the statistical analysis been performed appropriately and rigorously?

Yes, the analysis process is clear and rigorous, but I think it could be more concise and have a clearer focus. The current statistical analysis is not focused on answering a question, but demonstrates whatever the data could show, this should be further improved to the theoretical considerations.

*3. Have the authors made all data underlying the findings in their manuscript fully available?

Partly. The questionnaire questions are not fully included, and a demonstration of the kiosk collection machine or process would be helpful. And the presentation of certain tables are not fully clear, e.g. table 1 with unrecognized symbols. Figures in the appendix are rather difficult to follow.

*4. Is the manuscript presented in an intelligible fashion and written in standard English?

Partly. There are two major issues I think the authors could consider to improve. One is to unify the font of the article, the current line 577-589 is clearly not aligned with the rest. The other issue is the writing structure. The current article structure starts with introduction, but it feels more like a simplified literature review, the methods part could be compressed and add more about the justification on the methods used and the “methodological considerations” should be in this part instead of adding at the end, and please be aware, if you are not going to include the discussion of certain research design, in your case, the count of the audience applause and the monitor of CO2, please do not include it in the article. Overall, I think the manuscript writing significantly lacks academic indepth and the context of the research. I suggest the authors to re-work on the structure, and I think a clear research question would help.

Reviewer #3: This manuscript presents an interesting and well-designed study examining audience experience across repeated performances of the same theatre production using a mixed-methods approach. The integration of real-time kiosk data, questionnaire responses, and qualitative material is a clear strength, as is the relatively large sample size.

The study is technically sound overall, and the data broadly support the conclusions. However, several conceptual and methodological issues should be addressed to strengthen the manuscript.

First, the use of the term “identical performances” is conceptually misleading. While the production elements remain constant, the authors themselves acknowledge that audience composition, interaction, and contextual factors vary across performances. Given the co-constructed and dynamic nature of live theatre, this terminology should be clarified, as the performances are comparable but not truly identical.

Second, the interpretation of variability across performances would benefit from a more explicit consideration of audience composition. Since demographic data were collected only for a subset of participants and appear skewed (e.g., toward older age groups), it is difficult to determine whether observed differences reflect properties of the performance event or differences in the audience. This limitation should be more clearly acknowledged.

Third, the concept of “collective audience experience” would benefit from stronger theoretical grounding. At present, it is primarily operationalized through aggregated ratings, and a clearer conceptual definition would enhance the interpretation of the findings.

Fourth, while the statistical analyses are appropriate, they are largely descriptive. The cluster analysis identifies meaningful groupings; however, the interpretation of these clusters remains relatively straightforward. A more cautious framing or further elaboration would strengthen this part of the manuscript.

Finally, some questionnaire items appear conceptually overlapping (e.g., enjoyment, engagement, experience), as the authors also note. Further clarification or streamlining of the instrument would improve its conceptual clarity.

Overall, this is a valuable and methodologically interesting contribution, particularly in its design and data collection approach. Addressing the points above would substantially improve the clarity and interpretability of the manuscript.

**********

what does this mean?). If published, this will include your full peer review and any attached files.

If you choose “no”, your identity will remain anonymous but your review may still be made public.

**Do you want your identity to be public for this peer review?**  For information about this choice, including consent withdrawal, please see our Privacy Policy

Reviewer #1: **Yes:**  Hendi Sugianto

Reviewer #2: No

Reviewer #3: No

**********

[NOTE: If reviewer comments were submitted as an attachment file, they will be attached to this email and accessible via the submission site. Please log into your account, locate the manuscript record, and check for the action link "View Attachments". If this link does not appear, there are no attachment files.]

To ensure your figures meet our technical requirements, please review our figure guidelines: https://journals.plos.org/plosone/s/figures

You may also use PLOS’s free figure tool, NAAS, to help you prepare publication quality figures: https://journals.plos.org/plosone/s/figures#loc-tools-for-figure-preparation.

NAAS will assess whether your figures meet our technical requirements by comparing each figure against our figure specifications.

# Author comment: Author response to Decision Letter 1

**DOI:** 10.1371/journal.pone.0344225.r002

1 Sep 2026

All is in the attached rebuttal.

Attachment Submitted filename: Response_to_Reviewers.docx

# Peer review: Decision Letter 1

**DOI:** 10.1371/journal.pone.0344225.r003

**Authors:** Rachel Chan

24 Sep 2026

Measuring audience experience across 20 showings of the same theatre performance using customer feedback kiosks and a questionnaire

PONE-D-25-66243R1

Dear Dr. Sams,

We’re pleased to inform you that your manuscript has been judged scientifically suitable for publication and will be formally accepted for publication once it meets all outstanding technical requirements.

Within one week, you’ll receive an e-mail detailing the required amendments. When these have been addressed, you’ll receive a formal acceptance letter and your manuscript will be scheduled for publication.

An invoice will be generated when your article is formally accepted. Please note, if your institution has a publishing partnership with PLOS and your article meets the relevant criteria, all or part of your publication costs will be covered. Please make sure your user information is up-to-date by logging into Editorial Manager at Editorial Manager® and clicking the ‘Update My Information' link at the top of the page. For questions related to billing, please contact billing support.

If your institution or institutions have a press office, please notify them about your upcoming paper to help maximize its impact. If they’ll be preparing press materials, please inform our press team as soon as possible -- no later than 48 hours after receiving the formal acceptance. Your manuscript will remain under strict press embargo until 2 pm Eastern Time on the date of publication. For more information, please contact onepress@plos.org.

Kind regards,

Rachel Suet Kay Chan, Ph.D.

Academic Editor

PLOS One

Additional Editor Comments (optional):

Reviewers' comments:

Reviewer's Responses to Questions

**Comments to the Author**

Reviewer #1: All comments have been addressed

Reviewer #2: All comments have been addressed

**********

2. Is the manuscript technically sound, and do the data support the conclusions?

Reviewer #1: Yes

Reviewer #2: Yes

**********

3. Has the statistical analysis been performed appropriately and rigorously?

Reviewer #1: Yes

Reviewer #2: Yes

**********

4. Have the authors made all data underlying the findings in their manuscript fully available?

The PLOS Data policy

Reviewer #1: Yes

Reviewer #2: Yes

**********

5. Is the manuscript presented in an intelligible fashion and written in standard English?

Reviewer #1: Yes

Reviewer #2: Yes

**********

Reviewer #1: The authors have adequately addressed all my previous comments, including the typo corrections and the clarification of the consent and questionnaire administration procedure in the Methods (p. 7). The statistical methods used are appropriate and rigorously applied for this study design. I have no further concerns and recommend acceptance.

Reviewer #2: All comments have been addressed thoroughly. The clearly stated research question provides strong structure for the implementation and discussion sections. Furthermore, the revised methodology section offers valuable depth regarding the data collection process, particularly in comparing the kiosk and questionnaire data. Overall, this manuscript is significantly improved from the initial submission.

**********

what does this mean?). If published, this will include your full peer review and any attached files.

If you choose “no”, your identity will remain anonymous but your review may still be made public.

**Do you want your identity to be public for this peer review?**  For information about this choice, including consent withdrawal, please see our Privacy Policy

Reviewer #1: No

Reviewer #2: No

**********

# Editor report: Acceptance letter

**DOI:** 10.1371/journal.pone.0344225.r004

**Authors:** Rachel Chan

PONE-D-25-66243R1

PLOS One

Dear Dr. Sams,

I'm pleased to inform you that your manuscript has been deemed suitable for publication in PLOS One. Congratulations! Your manuscript is now being handed over to our production team.

At this stage, our production department will prepare your paper for publication. This includes ensuring the following:

* All references, tables, and figures are properly cited

* All relevant supporting information is included in the manuscript submission,

* There are no issues that prevent the paper from being properly typeset

You will receive further instructions from the production team, including instructions on how to review your proof when it is ready. Please keep in mind that we are working through a large volume of accepted articles, so please give us a few days to review your paper and let you know the next and final steps.

Lastly, if your institution or institutions have a press office, please let them know about your upcoming paper now to help maximize its impact. If they'll be preparing press materials, please inform our press team within the next 48 hours. Your manuscript will remain under strict press embargo until 2 pm Eastern Time on the date of publication. For more information, please contact onepress@plos.org.

You will receive an invoice from PLOS for your publication fee after your manuscript has reached the completed accept phase. If you receive an email requesting payment before acceptance or for any other service, this may be a phishing scheme. Learn how to identify phishing emails and protect your accounts at https://explore.plos.org/phishing.

If we can help with anything else, please email us at customercare@plos.org.

Thank you for submitting your work to PLOS One and supporting open access.

Kind regards,

PLOS One Editorial Office Staff

on behalf of

Dr. Rachel Suet Kay Chan

Academic Editor

PLOS One
